# Global reach of ageism on older persons’ health: A systematic review

**DOI:** 10.1371/journal.pone.0220857

**Published:** 2020-01-15

**Authors:** E-Shien Chang, Sneha Kannoth, Samantha Levy, Shi-Yi Wang, John E. Lee, Becca R. Levy

**Affiliations:** 1 Department of Social and Behavioral Sciences, Yale School of Public Health, New Haven, Connecticut, United States of America; 2 Department of Chronic Disease Epidemiology, Yale School of Public Health, New Haven, Connecticut, United States of America; 3 Department of Internal Medicine, Yale School of Medicine, New Haven, Connecticut, United States of America; 4 Department of Psychology, Yale University, New Haven, Connecticut, United States of America; Cardiff University, UNITED KINGDOM

## Abstract

**Objective:**

Although there is anecdotal evidence of ageism occurring at both the structural level (in which societal institutions reinforce systematic bias against older persons) and individual level (in which older persons take in the negative views of aging of their culture), previous systematic reviews have not examined how both levels simultaneously influence health. Thus, the impact of ageism may be underestimated. We hypothesized that a comprehensive systematic review would reveal that these ageism levels adversely impact the health of older persons across geography, health outcomes, and time.

**Method:**

A literature search was performed using 14 databases with no restrictions on region, language, and publication type. The systematic search yielded 13,691 papers for screening, 638 for full review, and 422 studies for analyses. Sensitivity analyses that adjusted for sample size and study quality were conducted using standardized tools. The study protocol is registered (PROSPERO CRD42018090857).

**Results:**

Ageism led to significantly worse health outcomes in 95.5% of the studies and 74.0% of the 1,159 ageism-health associations examined. The studies reported ageism effects in all 45 countries, 11 health domains, and 25 years studied, with the prevalence of significant findings increasing over time (p < .0001). A greater prevalence of significant ageism-health findings was found in less-developed countries than more-developed countries (p = .0002). Older persons who were less educated were particularly likely to experience adverse health effects of ageism. Evidence of ageism was found across the age, sex, and race/ethnicity of the targeters (i.e., persons perpetrating ageism).

**Conclusion:**

The current analysis which included over 7 million participants is the most comprehensive review of health consequences of ageism to date. Considering that the analysis revealed that the detrimental impact of ageism on older persons’ health has been occurring simultaneously at the structural and individual level in five continents, our systematic review demonstrates the pernicious reach of ageism.

## Introduction

To understand health inequities, it is important to consider how upstream, or structural, factors trickle down to influence the health of individuals. This approach has been used to examine health inequities due to social class [1–4], as well as due to prejudice based on race [5], sexual orientation and gender identity [6, 7]. It has also been postulated that health inequities among older persons due to ageism can operate at both the structural level (i.e., in which societal institutions promote bias against older persons) and the individual level (i.e., in which older persons assimilate negative beliefs about aging from their culture) [8–10]. Yet, previous ageism studies, and the reviews based on them, have focused on the consequences of ageism operating at the individual level, without assessing the structural sources of ageism. For example, the impact of individual-level ageism has been highlighted by five meta-analyses that showed negative age beliefs can adversely affect the health of older persons [11–15].

Further, previous reviews that have documented ageism at the structural level have not simultaneously assessed how this impacts the health of older persons [16, 17]. To illustrate, the exclusion of older persons from medical treatments has been studied, but without considering the health consequences on older persons [18]. Consequently, previous structural level and individual level studies may have underestimated the damaging effect of ageism on health.

Our current study draws on stereotype embodiment theory (SET) [10], which explains how three distinct, yet interrelated, components of ageism can impact health: age discrimination (i.e., detrimental treatment of older persons); negative age stereotypes (i.e., beliefs about older persons in general); and negative self-perceptions of aging (i.e., beliefs held by older persons about their own aging). According to SET, these three components of ageism deleteriously influence the health of older persons through psychological, behavioral, and physiological pathways [10].

In the current study, the term “individual ageism” includes the impact of culture-based negative age stereotypes and negative self-perceptions of aging on the health of older persons. “Structural ageism” refers to the explicit or implicit policies, practices, or procedures of societal institutions that discriminate against older persons; it can also include the age-based actions of individuals who are part of these institutions, such as the staff of a hospital [19, 20].

To assess the pernicious effect of ageism, the current study examined, for the first time, a wide array of studies that enable a global analysis of the impact that both structural- and individual-level ageism have on older persons in multiple health domains. The structural-level and individual-level ageism are inextricably linked because disparaging views of aging, that are propagated by word and deed at the structural level, are assimilated at the individual level [10].

In the following study, we hypothesized that: (1) ageism will adversely impact a broad range of health outcomes among older persons; and (2) this harmful pattern will exist regardless of geography and time as well as characteristics of the studies, targets, and targeters.

## Materials and methods

### Search strategy and inclusion criteria

This systematic review was conducted according to the Preferred Reporting Items for Systematic Reviews and Meta-Analyses (PRISMA) guidelines (see S1 Table for complete PRISMA checklist) [21]. As the priority during the screening stage was to capture all ageism-relevant studies, a detailed search strategy was created to include as many search terms as possible related to ageism (i.e., our predictor). In consultation with our team, data specialists at WHO developed a comprehensive list of “ageism” search terms. The search strategy combined key terms related to “ageism,” “age discrimination,” “age stereotype,” or “perceptions of aging” with terms related to “elder” or “older adults.” The terms were subsequently adapted to the search using 14 databases that contained both peer-reviewed articles and grey literature (i.e., conference proceedings and dissertations) that appeared after 1969, when ageism was first named [8], until December 2017. These databases included PubMed, PsycINFO, Ageline, EBSCO, Embase, CINAHL, Global Index Medicus, DARE, Epistemonikos, Cochrane Database of Systematic Reviews, Campbell Collaboration, Prospero, Greylit and Opengrey (see S2 Table for a list of search terms). Using this search procedure, the WHO team retrieved 21,379 citations. The total number of citations were reduced to 13,691 after removing duplicated records.

We included studies that: (1) quantitatively analyzed the effects of ageism on health, (2) controlled for age and other relevant covariates, or used an age-matched design, and (3) examined ageism targets aged 50 years or older. This age group was selected to be consistent with (1) population-based studies of aging globally that recruit participants aged 50 years and older [22–24], and (2) older workers faced with structural ageism are typically considered to be aged 50 years and older [25]. Studies that analyzed the unjust exclusion of older persons aged 50 years and older from clinical trials at the aggregate level were also included in our review. As the individual clinical trials included in these aggregate analyses did not inspect trends of age exclusion, we did not also include the individual clinical trials.

We used the WHO definition of health, which incorporates structural-level social environments that can influence it, such as the workplace and health care settings [26, 27].

Based on these inclusion criteria, two investigators then independently screened all 13,691 titles and abstracts, using Covidence, a systematic review management software (Veritas Health Innovation Ltd, Melbourne, Australia). Conflicts were resolved through a third reviewer via consensus.

In the initial stage of screening, two independent reviewers checked each study title and abstract to see if they met our inclusion criteria. Most of the studies that were excluded in the initial stage did not examine any ageism-health association (e.g., examined portrayals of ageism in children’s books). This led to identifying 638 studies for further full-text eligibility assessment. An additional two investigators appraised the full text of these studies. Again, any conflicts were resolved through a third reviewer via consensus, yielding 259 articles that met inclusion criteria. Out of studies excluded, 331 articles were not based on quantitative research that included ageism predictor on health outcome; 37 articles were missing statistics; 11 were duplications, such that the identical article was included both as grey-literature and peer-reviewed article. In cases of duplications, we selected the peer-reviewed article only. Studies that met any exclusion criteria were excluded.

**Fig 1:**
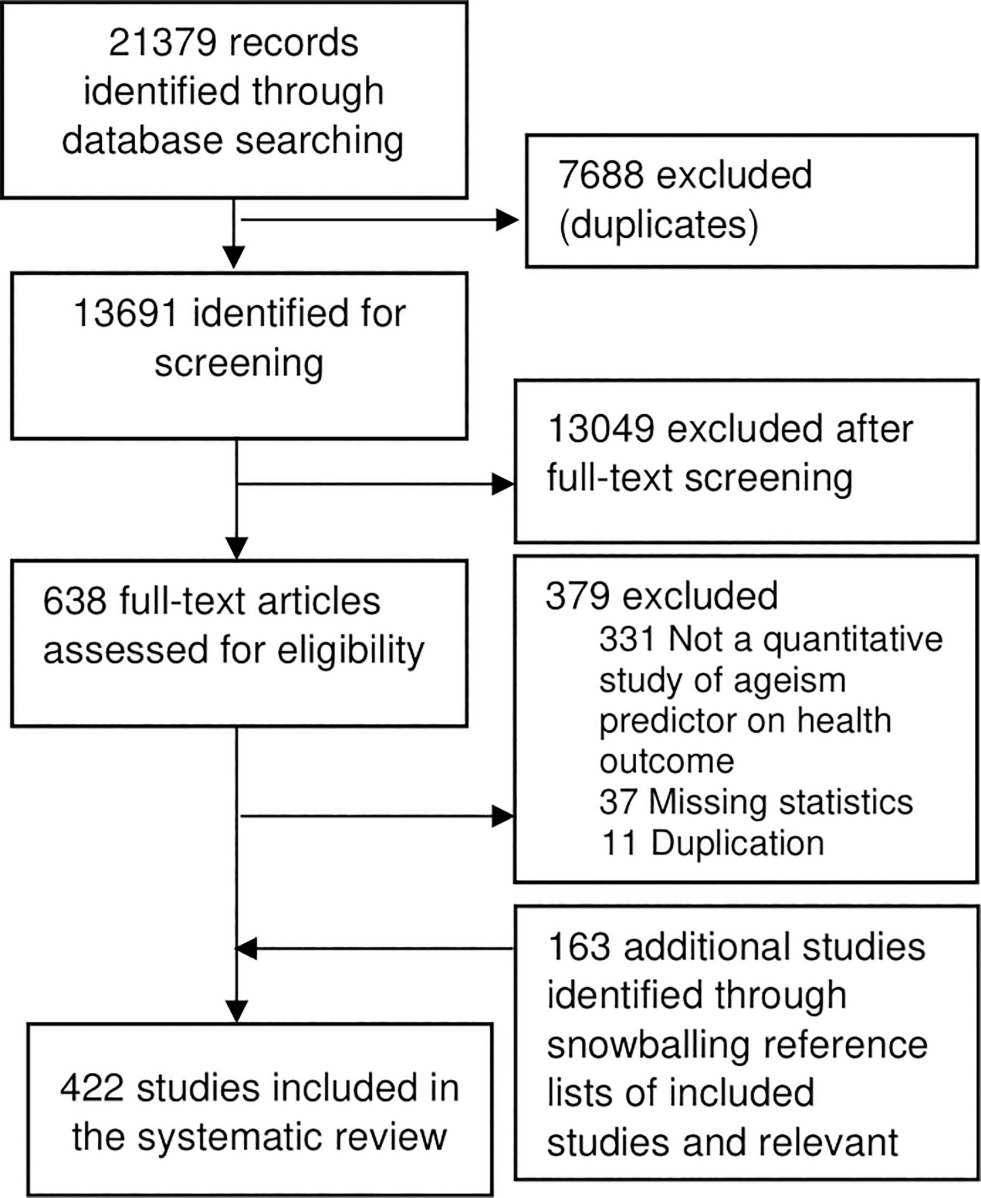
Flowchart of selection of ageism-health studies.

Two additional steps were employed to ensure we identified all relevant literature. First, we hand-searched through the reference lists of all relevant meta-analyses. Second, we used a snowballing technique and hand-searched the reference lists of all included articles in the final systematic review. Full texts were checked of all potentially relevant new titles. As a result, an additional 163 articles were identified and added to the final systematic review, leading to 422 studies (see Fig 1 for selection diagram and S3 Table for a full list of included studies).

The measures that were used in this review’s studies for assessing age discrimination included the Everyday Discrimination Scale [28] which measures how often participants experienced unequal treatments due to age; for assessing negative-age-stereotype included the Expectations Regarding Aging Survey [29]; and for assessing negative self- perceptions of aging included the Attitude Toward Own Aging subscale of the Philadelphia Geriatric Center Morale Scale [30, 31].

### Data extraction

Data on study characteristics, methods, and findings were independently extracted by two investigators. A data-extraction Microsoft Excel spreadsheet was developed and pilot-tested on ten randomly-selected studies to ensure adequacy and exhaustiveness of the tool. Three main categories of data were extracted: characteristics of the study samples relating to targets and targeters, methodological characteristics of each study, and results.

To generate the health categories that fit our health definition, a thematic analysis was conducted [32]. First, each health-outcome variable from the studies was recorded. Second, variables were grouped with the goal of identifying major nonoverlapping themes. Third, the themes were then reviewed independently by at least two investigators [33]. Any disagreements were resolved by discussions with a third investigator until a consensus was reached.

This process led to identifying four structural health domains inherent in the operation of social institutions or organizations, including (1) denied access to health care and treatments, (2) exclusion from clinical trials, (3) devalued lives (as assessed by age-rationing of social resources), and (4) lack of work opportunities. Additionally, we identified seven individual health domains including: (1) reduced longevity, (2) poor quality of life, (3) poor social relationships, (4) risky health behaviors, (5) mental illness, (6) cognitive impairment (as assessed by cognition over time), and (7) physical illness.

To be conservative, when studies included multiple outcomes, we abstracted information related to all of the outcomes, even when these outcomes were considered secondary and thus less likely to be powered to pick up on a significant result. We also determined whether each study reported at least one significant association between ageism and health, and whether the predicted association between ageism and worse health was significant at p < .05. As might be expected, none of the studies or associations identified in the review showed a significant association between ageism and better health. The data extraction revealed that of the 422 studies included in the review, most were: conducted in North America or Europe (78.2%), published since 2000 (81.5%), observational studies (68.3%) and published as peer-reviewed articles (91.5%) (see S4 Table for descriptions of 422 study characteristics).

### Quality appraisal

All included studies, both peer-reviewed publications and grey literature, were independently assessed on quality by two reviewers. Any discrepancies were resolved by a third reviewer.

For observational studies, we used the Newcastle-Ottawa quality assessment [34] to assess three broad areas: (1) the selection of the study groups, (2) the comparability of the groups, and (3) the ascertainment of either the exposure for case-control studies or the outcome of interest for cohort studies. This quality assessment tool is recommended for evaluating observational studies from the Cochrane Collaboration [35].

For experimental studies, we used the Downs and Black checklist to assess quality because it was developed specifically for appraising both randomized and non-randomized experimental studies [36]. The checklist evaluated domains including external validity, blinding, outcome data reporting, and reporting bias. This quality appraisal tool is also commonly used to evaluate health care research studies [37]. All items of each appraisal tool were summed to produce an overall score of 0–9, with a higher score indicating better quality.

### Data analysis

Data that met all inclusion criteria were first summarized descriptively, and then analyzed statistically. Analyses were conducted at both study-level and association-level, such that the prevalence of significant studies and associations for health domains, geography, time, study characteristics, and characteristics of the targeters and targets were examined.

To examine the first hypothesis that ageism will adversely impact a broad range of health outcomes among older persons, we first examined evidence of ageism by examining whether significant ageism-health findings emerged across different outcome domains. Evidence of ageism was defined as finding more than 50% of ageism-health associations as significant. To examine the second hypothesis that this harmful pattern will exist regardless of geography and time, as well as characteristics of the studies, targets, and targeters, we first examined whether significant ageism-health findings emerged in each country, year, and characteristic of the targets, and targeters. Similarly, evidence of ageism was defined as finding more than 50% of ageism-health associations as significant. We then performed bivariate analyses using chi-squared tests to examine patterns of ageism by geography, time, characteristics of study, publication type (peer-reviewed articles vs grey literature), targets, and targeters.

To examine the robustness of the findings, we performed two sensitivity analyses. For the first sensitivity analysis, we repeated the aforementioned analyses in a subset of studies that was appraised as good-quality studies with overall score of 7 and above (n = 317; 75.1%), based on our study-quality-appraisal checklists [34, 36]. For the second sensitivity analysis, we conducted a multivariate logistic regression to control for study size in the full set of studies, as well as in the subset of good-quality studies.

As a secondary analysis, we estimated the number of global cases of depression due to ageism among older persons in one year. We examined depression because it was the health condition with the largest number of studies (n = 17) that had the most homogenous outcome measure; 82.4% (n = 14) used the Center for Epidemiologic Studies Depression Scale (CESD) [38]. To compute the number of global cases of depression due to ageism among older persons, following a previously developed model [11], we calculated the number of older persons aged 50 and over in the high-ageism groups, as well as the difference in the rates of depression for those in the low- and high-ageism groups. To understand ageism patterns globally, we computed the numbers of cases separately in the more- and less-developed countries in 2015. See S1 Appendix for the descriptions of the sources and calculations used to estimate the global depression cases due to ageism among older persons.

All analyses were conducted in SAS (version 9.4, SAS Institute Inc., Cary, NC) and STATA (version 13.1, StataCorp, College Station, TX).

## Results

### Impact of ageism on health of older persons: Structural level

As predicted by the first hypothesis, ageism was found to adversely impact a broad range of health outcomes among older persons: 95.5% of the 422 studies and 74.0% of the 1,159 associations between ageism and health showed evidence of the adverse effects of ageism. Ageism was significantly related to worse health in all 11 health domains (Table 1). As hypothesized, evidence of ageism was found across all health domains; the proportion of significant ageism-health associations ranged from 94.4% to 63.0% in 11 health domains.

**Table 1:** Evidence of ageism as indicated by significant studies and significant associations across 11 health domains^a^.

	Significant Ageism Studies	Significant Ageism Associations
	% (n)^b^^,^^c^	% (n)
**Exclusion from health research**	100.0 (49)	94.4 (70)
**Devalued lives of older persons**	100.0 (3)	80.0 (4)
**Lack-of-work opportunities**	89.6 (60)	80.8 (97)
**Denied access to healthcare and treatments**	84.6 (141)	63.0 (376)
**Reduced longevity**	100.0 (10)	85.7 (12)
**Poor quality-of-life and well-being**	100.0 (29)	93.3 (42)
**Risky health behaviors**	100.0 (27)	79.4 (50)
**Poor social relationships**	100.0 (13)	82.5 (47)
**Physical illness**	96.2 (50)	80.9 (72)
**Mental illness**	95.5 (42)	93.2 (82)
**Cognitive impairment**	80.0 (4)	85.7 (6)

^a^ As some studies included multiple outcomes, the total number of studies exceeded the number of articles included in the review. Percentages may not sum to 100% due to rounding.

^b^ All ageism studies and associations in the review either showed a significant effect in the predicted direction with ageism predicting worse health or showed no significant association. None of the studies showed the reverse direction with ageism significantly predicting better health. Thus, in the current table, we presented the percentage of the studies or associations in each domain that were significant in the predicted direction and give the total number of studies to give overview. For instance, in the mental illness category, there were 82 predicted significant associations between ageism and worse health, which was 93.2% of the total 88 associations in this category. The remaining 6 associations (88–82 = 6) did not reach significance.

^c^ Significant ageism studies are defined as studies that showed at least one significant association at the p < .05 level between ageism and adverse-health outcome. Significant association between ageism and adverse health is defined as the p < .05 level.

**Fig 2:**
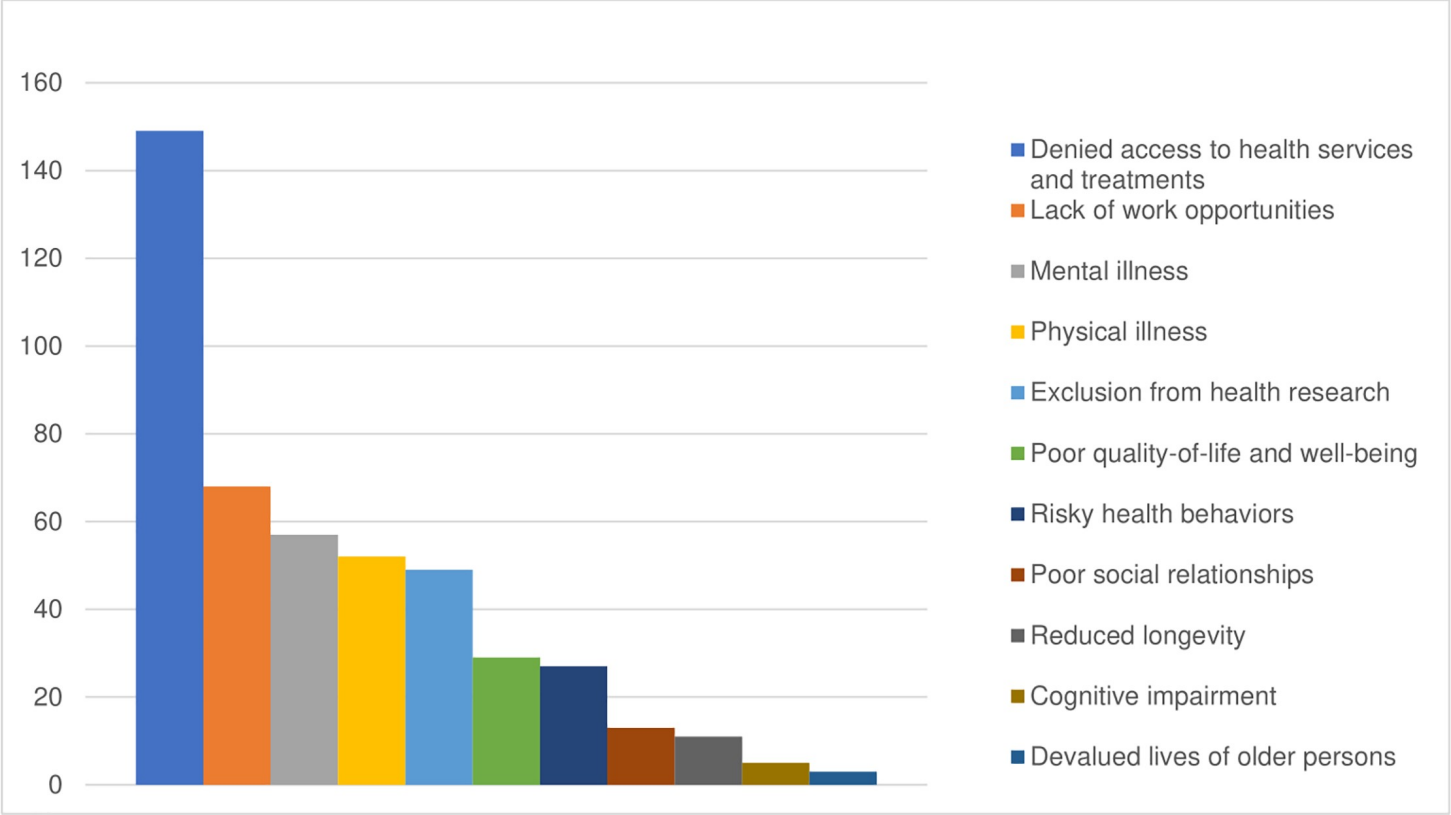
Impact of ageism on health in 11 health domains: Distribution of studies.

At the structural level, all four health domains showed evidence of ageism. Denied access to health services and treatments was the most researched aspect of structural ageism, with 149 studies and 545 associations (Fig 2). In 84.6% of the studies and 63.0% of the associations, age dictated whom receives certain procedures or treatments. For example, in a study of 9,105 hospitalized patients, health care providers were significantly more likely to withhold life-sustaining treatments from older patients, compared to younger ones, after controlling for patients’ prognosis and care preferences [39].

In the domain of older persons’ exclusion from health research, all 49 studies and 94.4% of the 74 associations showed evidence of ageism. These studies showed that older persons were excluded from trials in cardiology, internal medicine, nephrology, neurology, preventive medicine, psychiatry, rheumatology, oncology, and urology [40–46]. Most of these studies were based on global trial-registry data (81.7%) which included up to 206 countries [47]. For example, using an international registry of Parkinson’s disease clinical trials, researchers found 49.0% of the clinical trials excluded older persons, even though this disease is more prevalent in later life [48].

In the devalued-lives domain, 80.0% of the four associations showed evidence of ageism. For instance, Japanese participants were significantly more likely to sacrifice elderly pedestrians than younger pedestrians using run-away-trolley vignettes [49]. This category also included studies that found ageism contributed to age-rationing of treatments [50, 51].

In the lack-of-work-opportunities domain, 91.2% of the 34 associations in 27 studies found workplace ageism predicted worse health, such as increased depressive symptoms [52, 53] and long-term illness [54]. Older persons faced ageism throughout the employment-cycle stages. For example, 90.9% of the 22 associations revealed that employers were significantly less likely to hire older than younger job applicants. Once employed, older workers had less access to training (78.6% of the 14 associations) and those who faced ageism in the workplace were more likely to retire early (61.5% of the 13 associations). These studies included both blue- and white-collar workers living in 17 countries and four continents. For instance, British and American employers were significantly more likely to put older workers than younger workers with similar qualifications in positions with low pay and low responsibility [55].

### Impact of ageism on health of older persons: Individual level

In further support of the first hypothesis, ageism was also significantly associated with all seven health domains operating at the individual level. In four of these domains, all studies showed evidence of ageism. To illustrate, ageism consistently impacted the ultimate endpoint—reduced longevity: all 10 studies found ageism predicted a shorter lifespan in Australia, China, Germany, and the United States [56–65]. In one study, using nationally representative data in China, researchers found that older persons with more negative self-perceptions of aging had significantly reduced longevity [62].

In the poor-quality-of-life domain, all of the 29 studies and 93.9% of the 45 associations found evidence of ageism. For example, negative self-perceptions of aging predicted worse quality-of-life among socio-economically disadvantaged older Turkish women [66].

In the poor-social-relationship domain, all of the 13 studies and 82.5% of the 57 associations showed evidence of ageism. Outcomes included low social support, poor social engagement, and social isolation. To illustrate, the negative self-perceptions of aging held by Chinese older persons were significantly associated with dissatisfaction in social support provided by children [67].

In the risky-health-behavior domain, all 27 studies and 79.4% of the 63 associations showed evidence of ageism. Outcomes in this category included unhealthy diet, medication noncompliance, excessive drinking, and smoking. Results from a sample of 6,576 Irish older persons found negative self-perceptions of aging predicted increased risk of harmful drinking and smoking [68].

In the mental-illness domain (outside of work environments), 95.5% of the 44 studies and 93.2% of the 88 associations found evidence of ageism influencing psychiatric conditions. The most frequently examined condition, depressive symptoms, showed evidence that ageism was associated with onset and lifetime depression, as well as increases in depressive symptoms over time [69–83]. Reflecting a hopeful finding in this area, researchers found that when older persons resisted negative age stereotypes, they were less likely to experience suicidal ideation, anxiety, and PTSD in a nationally representative sample of American veterans [84].

In the cognitive-impairment domain, 80.0% of the five studies and 85.7% of the seven associations showed evidence of ageism using prospective data. For example, negative age stereotypes predicted worse memory as much as 38 years later [85]. These cognitive-impairment studies, conducted in China, Germany, Ireland, and the United States [85–89], complement the numerous experimental studies that found ageism predicted the cognitive performance of older persons [90, 91], a conclusion supported by two meta-analyses [12,14].

In the physical-illness domain, 96.2% of the 52 studies and 80.9% of the 89 associations significantly predicted physical illness, as assessed by functional impairment, chronic conditions, acute-medical-events incidence, and hospitalizations. For instance, older persons with negative age stereotypes were 31.0% less likely to recover from severe disability than those with positive age stereotypes [92]. Older persons with negative self-perceptions of aging were significantly more likely to show functional decline in studies undertaken in Israel, United States, and Australia [93–95].

### Health impact of ageism across geography and time

**Fig 3:**
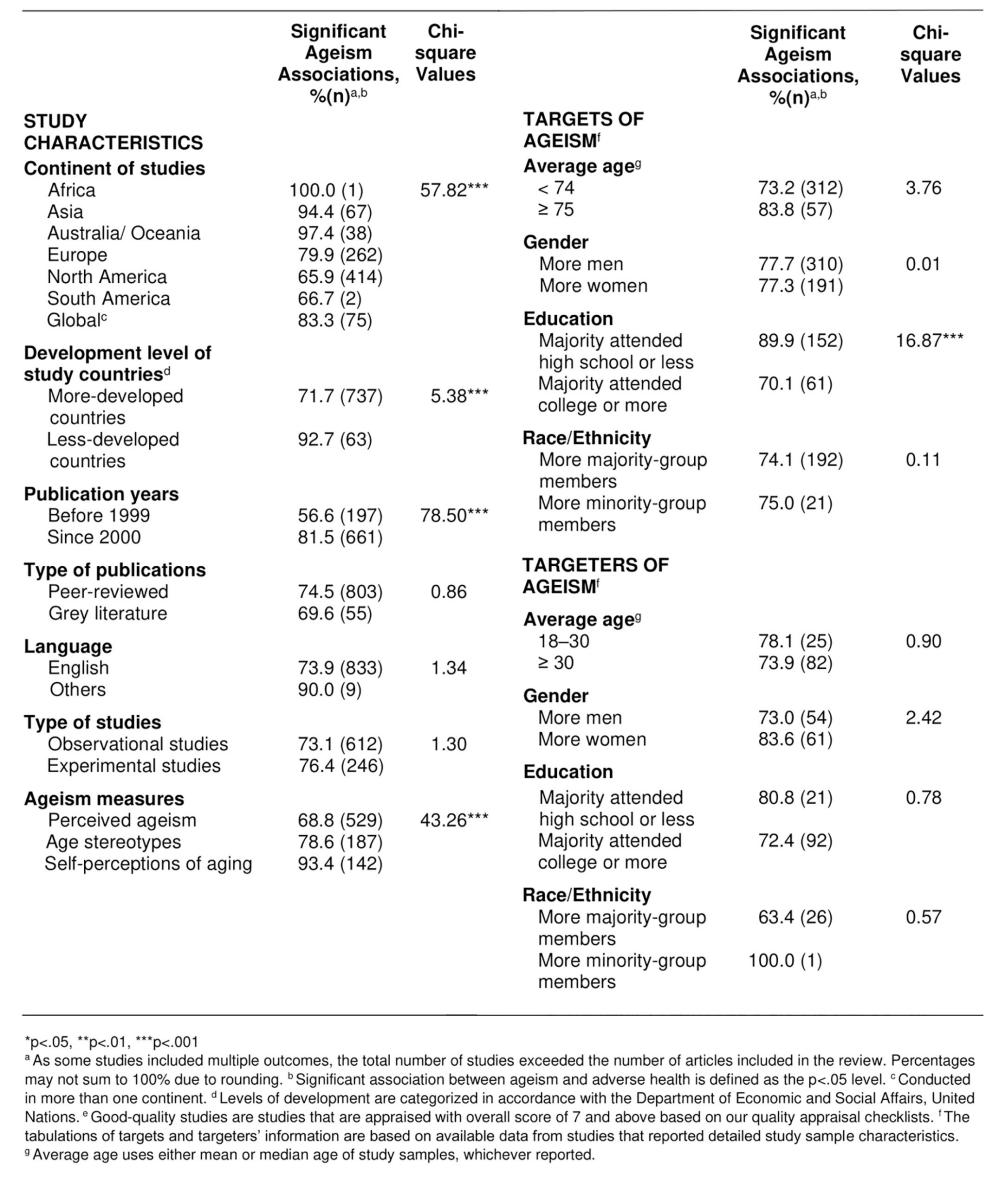
Ageism adversely impacted health across geography, time, and characteristics of studies, targets, and targeters.

**Fig 4:**
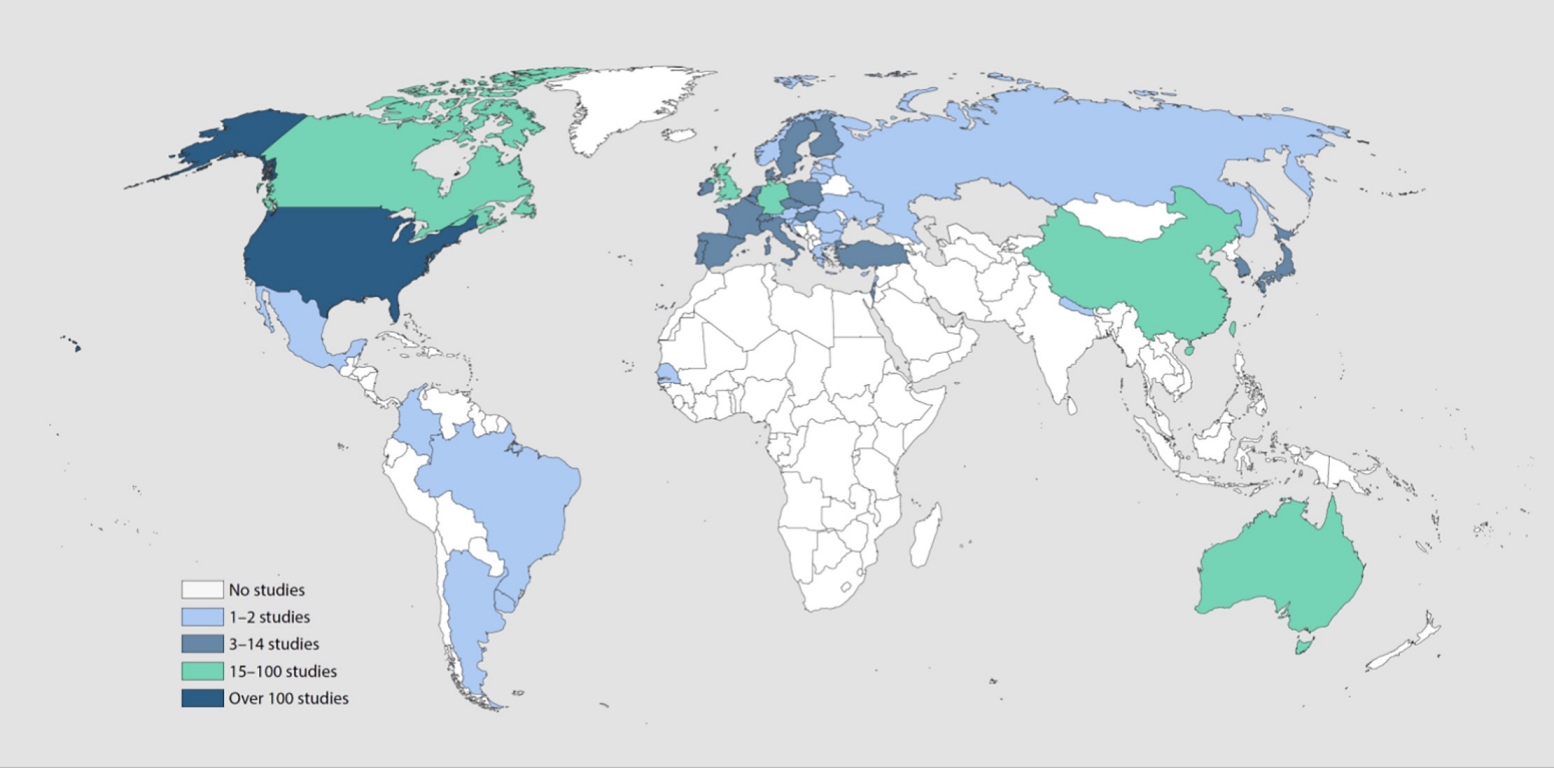
Geographic distribution of ageism studies across 45 countries.

As predicted by the second hypothesis, ageism adversely impacted health across geography and time (Fig 3). Ageism was observed in all 45 countries and all six continents in which studies took place (Fig 4). The prevalence of significant associations was higher in less-developed than more-developed countries (92.7% vs. 71.7%, p = .0002). Research conducted in Australia (97.4%) and Asia (94.4%) yielded more significant findings than in other continents (p < .0001). Only one study took place in Africa.

**Fig 5:**
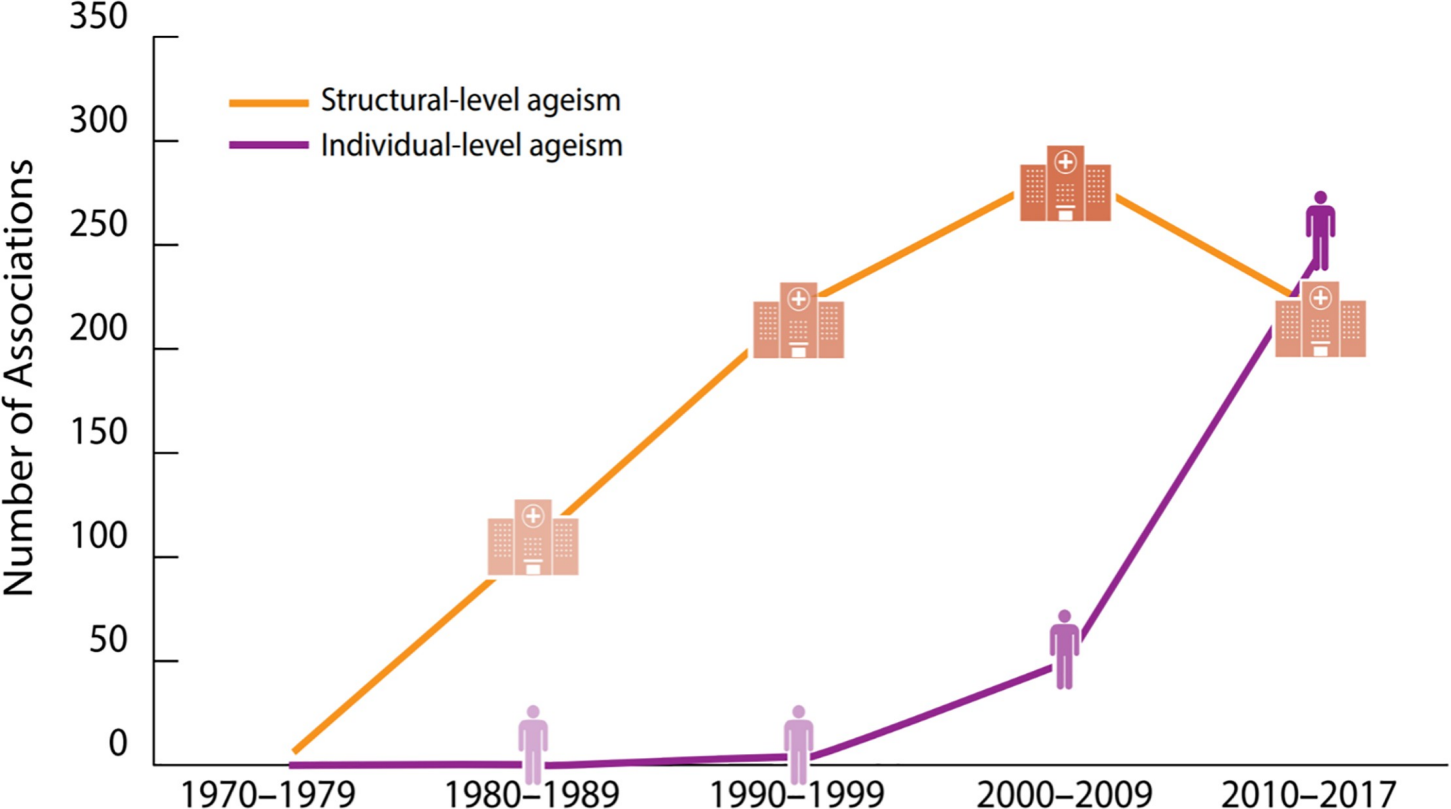
Research attention given to structural-level and individual-level ageism on health studies over time.

In addition, as predicted by the second hypothesis, ageism adversely impacted health across time. Ageism was observed in every year studied. Analyses also showed that the proportions of significant associations between ageism and adverse health in all 422 studies increased over time: 51.3% from 1970–1989, 69.6% from 1990–2009, and 85.3% from 2010–2017, p < .0001 (Fig 5). The proportion of significant findings for structural ageism also increased over time: 51.3% from 1970–1989, 66.8% from 1990–2009, 86.6% from 2010–2017, p < .0001. However, the number of associations addressing structural ageism pivoted during 2000–2009, when the trend of research examining these associations underwent a steady decline (Fig 5).

In terms of study characteristics, ageism was found to adversely impact health, regardless of the type of publication (peer-reviewed or grey literature) (p = .35), language of the study (p = .25), or study design (p = .25). In the structural ageism studies, the medium- and low-quality studies had a slightly larger proportion of significant findings compared with good-quality studies (p < .01). This may be due to the higher quality structural studies having more diverse samples. Studies in all quality levels exceeded the criterion of showing evidence of ageism-health associations.

Consistent with SET [10] and a recent analysis of the financial cost of ageism on health [11], we also found that the prevalence of significant ageism-health findings was highest when ageism was operationalized as self-perceptions of aging measure, compared with age stereotypes and perceived ageism measures (93.4%, 78.6%, 68.8% respectively; p < .0001).

### Health impact of ageism across characteristics of targets and targeters

Additionally, as predicted, evidence of ageism was found regardless of targets’ and targeters’ characteristics (i.e., age, sex, education, and racial/ethnic group membership) (Fig 3). Among targets, those with high-school-or-less education (89.8%) were significantly more likely to be targets of ageism than the more-educated group (70.1%, p < .0001).

For targeters, most studies of health care students and professionals found evidence of ageism (92.9%). Whereas the proportions of significant associations remained similar in other professions over time, the proportion of significant associations for health care professionals increased over time (p = .02 for trend). As an example, using vignettes of patients awaiting breast-cancer surgeries, a series of studies found that compared to younger patients with matched histories, older patients were significantly more likely to be denied treatment by new and advanced medical students as well as surgical and internal medicine residents [96–98].

Congruent with intersectionality theories that posit the converging health effects of multiple marginalizing characteristics [99, 100], minority racial/ethnic background can intensify the injurious effects of ageism. For instance, using matched-resume vignettes study (two identical job applicants with the exception of age and race), researchers in the U.K. found that not only older applicants were automatically sorted into lower-paid job vacancies compared to their younger counterparts, older applicants of minority racial/ethnic background were less likely to be interviewed compared with White older applicants [101].

In addition, we found that targets tended to be older than targeters, with a mean age of 66.4 and 34.0, respectively. Targets were less educated than targeters; the majority of targets had received high-school-or-less education (71.4%), whereas most targeters had received at least college education (73.3%) (see S4 Table description of the targets and targeters).

Our results from the sensitivity analyses replicated the patterns of significant results reported earlier. As in the overall sample of studies, when we only looked at higher quality studies and when we adjusted for study sample size, analyses revealed that ageism continued to adversely impacted health across the 11 domains, as well as across geography, time, study characteristics, and characteristics of the targets and targeters (see S5 Table and S6 Table for detailed sensitivity analyses).

As a secondary analysis to examine the number of global cases of depression attributable to ageism in older persons aged 50 and over, in our model we found there were 6.33 million cases of depression attributable to ageism with 831,041 cases in more-developed countries and 5.6 million cases in less-developed countries (see S1 Appendix for details of these calculations and for description of how the overlapping contribution of the predictors was removed from this ageism-depression estimate).

### Mediation of the association between ageism and health

To explore the mechanism by which ageism adversely impacted health in the included studies, we conducted a review to examine significant mediators. As more studies have demonstrated the ageism-health linkage, research has recently begun to identify the pathway by which ageism exerts an injurious effect on older persons’ health. In this review, 25 studies, represented eight countries, found statistically significant mediators that demonstrated pathways linking ageism to adverse health. The majority of the studies (79.6%) that investigated mediators between ageism and health were published since 2010.

Supporting SET [10], findings of mediation analyses indicate that ageism deleteriously influences the health of older persons through psychological, behavioral, and physiological pathways. Psychological pathway was supported by the most studies (9 longitudinal studies, 8 cross-sectional studies, and 2 experimental priming studies). To illustrate, studies found ageism could lead to adverse health outcomes due to the mediators of decreased levels of self-efficacy [95, 102–104], less perceived control [93] and purpose of life [74].

Researchers also explored the psychological mechanism between structural ageism and health, such as in the context of lack-of-work opportunities from the perspectives of both targets and targeters [105–108]. For instance, researchers found that German nurses who perceived higher ageist practice at work were less likely to identify with such organizational culture, which was then linked to higher intention to exit the workplace [107]. Similarly, other studies of ageism targets have found that the relationship between ageism and intentions for early retirement were mediated through negative job attitudes [108, 109].

The behavioral pathway between ageism and health was supported by four studies. Health-promoting behaviors in the form of physical activity was the most commonly investigated behavioral mediator between ageism and health [110–112]. That is, older persons who reported higher levels of ageism (i.e., negative age stereotypes and negative self-perceptions of aging) were less likely to engage in health-promoting behaviors, which, in turn, resulted in worse physical health. In the context of work, the association of older workers’ negative age stereotypes with an intention to retire sooner was mediated by reduced participation in career-enhancing activities [113].

The physiological pathway linking ageism to adverse health has only been examined by two studies so far. In one, a recent population-based study showed that the C-reactive protein, a stress biomarker of inflammation, partially mediated the longitudinal relationship between self-perceptions of aging and longevity [65].

## Discussion

In this first systematic review to include both structural- and individual-level studies, a strong and consistent link emerged between ageism and adverse health outcomes. Overall, in 95.5% of the 422 studies and in 74.0% of the 1,159 associations, ageism predicted significantly worse health outcomes, and impacted health in all of the countries studied.

Although significant associations between ageism and health were found in both less-developed and more-developed countries, the prevalence of these associations was significantly higher in the former than the latter. This finding does not indicate a higher level of ageism in the less-developed countries than the more-developed countries. In fact, among the less-developed countries, such as Nepal, there are often cultural traditions that promote positive-aging views [114]. The greater proportion of significant ageism-health associations in less-developed countries may, instead, be explained by their tending to have fewer resources to provide health care to older persons, compared to more-developed countries [115]. This potentially means that the stress generated among older persons by ageism [116] takes a greater toll on their health in less-developed countries because medical practitioners and facilities would be less able to mitigate the detrimental consequences of ageism than in more-developed countries. In addition, the pattern may be due in part to Confucianism and filial piety values leading to higher unmet expectations of respect in less-developed countries [117].

In line with prospective cohort and computational linguistics studies that found evidence of ageism increasing over time [118, 119, 120], the current study found that the proportion of significant findings related to structural-level ageism on health increased significantly over time. This significant increase of structural ageism-health findings after 2010 appears to coincide with the timing of the Great Recession. This pattern may reflect older adults’ particular vulnerabilities in labor force participation and financial well-being in economic downturns [121, 122]. It also parallels other types of prejudice and discrimination increasing during economic crises [123].

However, even though the prevalence of significant ageism-health associations has overall increased over time, research attention reflected in the number of studies have decreased in structural ageism studies. Specifically, with respect to research attention to studying ageism, the number of studies of individual-level ageism has increased significantly since the 1970s, whereas the number of structural-level ageism studies has decreased since 2010. This recent declining research interest in structural-level ageism limits a recognition of the central role that society plays in promoting and reinforcing discrimination towards older persons.

We also found that the prevalence of significant ageism-health findings was highest when ageism was operationalized by the self-perceptions of aging measure. This pattern is consistent with SET which postulates that self-relevance can increase the impact of ageism on health [10].

Our analysis of targets who are at most risk of ageism found older persons with lower levels of education were more likely to experience adverse health effects of ageism. This finding is in line with broader health-inequity literature that suggests members of disadvantaged social groups are more likely to become targets of discrimination [124]. This suggests that educational interventions to eradicate ageism might be especially effective [125].

This review also found a concerning trend of increasing ageism-health associations over time when health care professionals were targeters. This pattern echoes other reviews that revealed the growing practice of structural ageism by health care professionals. For instance, two recent systematic reviews showed that nurses’ as well as nurse-trainees’ attitudes toward older persons have grown more negative over the last decade [126, 127]. The increasing ageism-health associations in health care providers may be due to the growing time pressures: they are often required to see patients more quickly and to add the input of clinical information into electronic medical records as part of their daily tasks. Greater time pressure has been found to increase likelihood of stereotyping patients [128].

Our categorization of studies by levels of ageism and health outcomes revealed that ageism may operate at multiple reinforcing points. To illustrate, in the context of dementia on a structural level, older patients–particularly the oldest-old–remained disproportionally under-represented in clinical trials of Alzheimer’s disease [129–131]. Furthermore, during group or dyadic interactions, older persons with dementia were less likely to receive adequate assistance because of caregivers’ ageist communication styles [132, 133]. On an individual level, older persons who assimilated negative age stereotypes from society were more likely to develop Alzheimer’s-disease-related brain changes, compared to those who assimilated positive age stereotypes [134]. This, in turn, could compound encounters with structural ageism.

The reach of ageism was found to have clinical implications. First, ageism influenced a wide range of health outcomes of older patients. Second, vignette studies from eight countries showed that medical students and practitioners were apt to make clinical decisions that limited patients’ access to care based on patients’ age rather than their health needs [96–98, 135–158]. These decisions may have been made without awareness, insofar as studies have shown ageism can operate subliminally [90, 159]. Third, older persons were consistently denied inclusion in clinical trials of treatments, including those particularly relevant to them.

A recent report revealed the significant economic burden ageism imposes on society [11]. It was found that $63 billion, or one in every seven dollars, spent annually on American older persons’ health care for eight of the most expensive medical conditions was attributable to ageism [11]. In our current analysis, we also found that there are likely 6.33 million cases of older persons experiencing depression globally due to ageism. Thus, reducing ageism could not only improve the health of older persons; it could also be cost-effective. This could be especially valuable to less-developed countries which are expecting a large increase in the number and percentage of older citizens in upcoming decades [160].

Although our study was carried out to examine the impact of both structural and individual ageism on older persons’ health, this review has two limitations to note. First, due to the significant heterogeneity of the study outcomes, a meta-analysis was not feasible. Even with this heterogeneity, however, we found a consistent and strong association between ageism and a wide range of health outcomes. Second, qualitative studies were not included in the present review. Future reviews could integrate qualitative studies to provide additional insights into the ways in which ageism shapes health.

This systematic review highlights several additional gaps in the literature that could benefit from future research. First, our review found only four studies that examined ageism within dyads [132,133,161,162]. More research is needed on the ways in which ageism affects older individuals by seeping into the dynamic interactions of everyday life.

Second, in line with intersectional research [163,164], there is a need to better understand how intersecting systems of power reinforce ageism directed at marginalized groups within older populations. In the current review, few studies (n = 18) conducted formal statistical analyses to explore effect modifiers between ageism and adverse health. This is an important area for future research.

Third, our finding that in less-developed countries 92.7% of the ageism-health associations were significant, but only 8.6% of the studies were conducted there, suggests the need for additional ageism research in these regions. Cross-national comparative research may shed additional light on the political, economic, and contextual differences that contribute to differential impact of ageism experienced by older persons.

Fourth, future studies should also examine the relationship between structural and individual ageism. The ways in which structural ageism and individual ageism may jointly magnify health disadvantages warrant further examination.

In conclusion, the current findings underscore ageism as a social determinant of health. The significant adverse relationship between ageism and health in our review appears even more consistent than the relationships found in systematic reviews of racism’s effect on health [165,166]. Our findings showed that in 74.0% of the 1,159 associations, ageism predicted significantly worse health outcomes. Using comparable methodologies, systematic reviews have found that 41.6% - 64.2% of the associations were significant between racism and adverse health [165,166].

Initiatives to improve population health would benefit from taking ageism into account. By including all regions, languages, and health outcomes, our review included the most studies to date of the consequences of ageism. Accordingly, this review allows clinicians and policy makers to evaluate the consequences of ageism beyond national and regional borders and across time.

## Supporting information

S1 Table PRISMA checklist. (PDF)

S2 Table List of search terms. (PDF)

S3 Table Full list of included studies. (PDF)

S4 Table Characteristics of 422 Studies on ageism and health. (PDF)

S5 Table Sensitivity analysis of ageism adversely impacted health across geography, time, study characteristics, and characteristics of targets and targeters in good-quality studies. (PDF)

S6 Table Sensitivity analysis of predictors of significant ageism-health associations remained the same after adjusting for study sample size. (PDF)

S1 Appendix Description of the sources and calculations for computing the number of cases of depression due to ageism. (PDF)
