# Supplementary material for: Global reach of ageism on older persons’ health: A systematic review
Source: PLoS One. 2020 Jan 15;15(1):e0220857. doi: 10.1371/journal.pone.0220857 (PMC6961830; doi:10.1371/journal.pone.0220857)
Supplement: S2 Table — (PDF) [file pone.0220857.s002.pdf]

| S2 Table. List of Search Terms | |
|---|---|
| **Concept** | **Search string** |
| **1-Ageism** | (“Ageism”[MeSH] OR ageism[TiAb] OR agism[TiAb] OR ageist[TiAb] OR agist[TiAb] OR “age discrimination”[TiAb] OR “age prejudice”[TiAb] OR “age stereotype”[TiAb] OR “self perceptions of ageing”[TiAB] OR “self perceptions of aging”[TiAB] OR "age identity"[Tiab])<br>OR<br>(("Aged"[Mesh] OR “Aged, 80 and over”[MeSH] OR “Frail Elderly”[MeSH] OR elder*[TiAb] OR “seniors”[TiAb] OR “older adult”[TiAb] OR “older person”[TiAb] OR “older adults”[TiAb] OR “older persons”[TiAb] OR “older peoples”[tiab] OR “older people”[TiAb] OR “aging”[MeSH] OR “ageing”[TiAb] OR “aging”[TiAb] OR “Old age”[Ti]) AND ( "Social Exclusion"[Tiab] OR "social rejection"[TIab] OR "Social Acceptance"[TIab] OR “stereotyped behavior”[Mesh] OR “social perception”[Ti] OR “age identification”[ti] OR “self-perceptions”[tiAb] OR “Prejudice”[MeSH] OR “prejudice”[TiAb] OR stereotyp*[TiAb] OR “Stereotyping”[MeSH] OR "Social Discrimination"[Mesh] OR “Intergenerational Relations”[Mesh]))<br>NOT ( “Animals” NOT ("Animals"[Mesh] AND "Humans"[Mesh]))<br>NOT Plants[Mesh] |
