# Supplementary material for: Global reach of ageism on older persons’ health: A systematic review
Source: PLoS One. 2020 Jan 15;15(1):e0220857. doi: 10.1371/journal.pone.0220857 (PMC6961830; doi:10.1371/journal.pone.0220857)
Supplement: S3 Table — (PDF) [file pone.0220857.s003.pdf]

S3. Full List of Included Studies (n=422)

1. Abrams D, Eller A, Bryant J. Age apart: the effects of intergenerational contact and stereotype threat on performance and intergroup bias. Psychol Aging 2006 ; 21: 691-702.

2. Abrams D, Swift HJ, Drury L. Old and unemployable? How age-based stereotypes affect willingness to hire job candidates. J Social Issues 2016; 72: 105-21.

3. Acker ABT. The influence of ageism on personnel decision making. PhD thesis, University of Texas, Arlington, 2009.

4. Adams JN, Jamieson M, Rawles JM, Trent RJ, Jennings KP. Women and myocardial infarction: agism rather than sexism? Br Heart J 1995; 73: 87-91.

5. Adams SJ. Passed over for promotion because of age: an empirical analysis of the consequences. J of Labor Res. 2002; 23: 447-61.

6. Alcover C-M. ¿Ageism en las Organizaciones? El Papel Mediador del Apoyo Organizacional Percibido en las Relaciones entre la Edad y la Ruptura del Contrato Psicológico. [Ageism in Organizations? Perceived Organizational Support as a Mediator of the Relations Between Age and Psychological Contract Breach] Revista Psicologia Organizações e Trabalho 2012; 12: 299-313.

7. Alden EC, Gordon AR, Hernandez M, Olsson MJ, Lundstrom JN. Mind over age - social priming and olfactory function. Chem Senses 2009; 34: A24.

8. Alvarez-Galvez J, Salvador-Carulla L. Perceived discrimination and self-rated health in Europe: Evidence from the European Social Survey (2010). PLoS One 2013; 8: e74252.

9. Amoah DC. Self-perceptions as determinants of prescription medication compliance in the elderly. PhD thesis, United States International University, 1996.

10. Andersen S, Laurberg P. Age discrimination in osteoporosis screening - data from the Aalborg university hospital record for osteoporosis risk assessment (AURORA). Maturitas 2014; 77: 330-35.

11. Andrade SE, Majumdar SR, Chan KA, et al. Low frequency of treatment of osteoporosis among postmenopausal women following a fracture. Arch Intern Med 2003 ; 163: 2052-57.

12. Andrews RM, Tan EJ, Varma VR, et al. Positive aging expectations are associated with physical activity among urban-dwelling older adults. Gerontologist. 2017; 57S2: S178-86.

13. Arber S, McKinlay J, Adams A, Marceau L, Link C, O'Donnell A. Influence of patient characteristics on doctors' questioning and lifestyle advice for coronary heart disease: a UK/US video experiment. Br J Gen Pract 2004 ; 54: 673-8.

14. Arber S, McKinlay J, Adams A, Marceau L, Link C, O'Donnell A. Patient characteristics and inequalities in doctors' diagnostic and management strategies relating to CHD: a video-simulation experiment. Soc Sci Med 2006 ; 62: 103-15.

15. Arino A. Ageism and Coronary Care in Wales II. Age Ageing 1992; 21S2: 15.

16. Asahina Y, Sugano H, Sugiyama E, Uyama Y. Representation of older patients in clinical trials for drug approval in Japan. J Nutr Health Aging 2014; 18: 520-23.

17. Atkinson JL, Sloan RG. Exploring the impact of age, race, and stereotypes on perceptions of language performance and patronizing speech. J Lang Soc Psychol 2017; 36: 287-305.

18. Auman LC. Effect of stereotype threat on cognitive performance and physiological variability in older adults. PhD thesis, North Carolina State University, 2002.

19. Austin S, Qu H, Shewchuk RM. Age bias in physicians' recommendations for physical activity: A behavioral model of healthcare utilization for adults with arthritis. J Phys Act Health 2013; 10: 222-31.

20. Avezum A, Makdisse M, Spencer F, et al. Impact of age on management and outcome of acute coronary syndrome: observations from the Global Registry of Acute Coronary Events (GRACE). Am Heart J 2005; 149: 67-73.

21. Ayalon L. Perceived age discrimination: A precipitator or a consequence of depressive symptoms? J Gerontol B Psychol Sci Soc Sci 2016; 73: 860-69.

22. Ayalon L. Satisfaction with aging results in reduced risk for falling. Int Psychogeriatr 2016; 28: 741-47.

23. Ayalon L, Gum AM. The relationships between major lifetime discrimination, everyday discrimination, and mental health in three racial and ethnic groups of older adults. Aging Ment Health 2011; 15: 587-94.

24. Bagnall AJ, Goodman SG, Fox KA, et al. Influence of age on use of cardiac catheterization and associated outcomes in patients with non-ST-elevation acute coronary syndromes. Am J Cardiol 2009; 103: 1530-36.

25. Bai X, Lai DWL, Guo A. Ageism and depression: Perceptions of older people as a burden in China. J Soc Issues 2016; 72: 26-46.

26. Bailey C, Corner J, Addington-Hall J, Kumar D, Nelson M, Haviland J. Treatment decisions in older patients with colorectal cancer: the role of age and multidimensional function. Eur J Cancer Care 2003; 12: 257-62.

27. Bal PM, de Lange AH, Van der Heijden BIJM, Zacher H, Oderkerk FA, Otten S. Young at heart, old at work? Relations between age, (meta-)stereotypes, self-categorization, and retirement attitudes. J Vocat Behav 2015; 91: 35-45.

28. Ballard-Barbash R, Potosky AL, Harlan LC, Nayfield SG, Kessler LG. Factors associated with surgical and radiation therapy for early stage breast cancer in older women. J Natl Cancer Inst 1996; 88: 716-26.

29. Bandyopadhyay S, Bayer AJ, O'Mahony MS. Age and gender bias in statin trials. QJM 2001; 94: 127-32.

30. Banzi R, Camaioni P, Tettamanti M, Bertele V, Lucca U. Older patients are still under-represented in clinical trials of Alzheimer's disease. Alzheimers Res Ther 2016; 8: 32.

31. Barakat K, Wilkinson P, Deaner A, Fluck D, Ranjadayalan K, Timmis A. How should age affect management of acute myocardial infarction? A prospective cohort study. Lancet 1999; 353: 955-59.

32. Barber SJ, Lee SR. Stereotype threat lowers older adults' self-reported hearing abilities. Gerontology 2015; 62: 81-85.

33. Barker M, O'Hanlon A, McGee HM, Hickey A, Conroy RM. Cross-sectional validation of the aging perceptions questionnaire: a multidimensional instrument for assessing self-perceptions of aging. BMC Geriatr. 2007; 7: 9.

34. Barnow S, Linden M, Lucht M, Freyberger H-J. Influence of age of patients who wish to die on treatment decisions by physicians and nurses. Am J Geriatr Psychiatry 2004; 12: 258-64.

35. Bayl-Smith P, Griffin B. Age discrimination in the workplace: identifying as a late-career worker and its relationship with engagement and intended retirement age. J Appl Soc Psychol 2014; 44: 588-99.

36. Bearden D, Allman R, McDonald R, Miller S, Pressel S, Petrovitch H. Age, race, and gender variation in the utilization of coronary artery bypass surgery and angioplasty in SHEP. SHEP Cooperative Research Group. Systolic Hypertension in the Elderly Program. J Am Geriatr Soc 1994; 42: 1143-49.

37. Bearden DM, Allman RM, Sundarum SV, Burst NM, Bartolucci AA. Age-related variability in the use of cardiovascular imaging procedures. J Am Geriatr Soc 1993; 41: 1075-82.

38. Beckett P, Woolhouse I, Peake MD, Stanley R, Harrison RN. P210 The influence of age on management of lung cancer patients in England. Thorax 2010; 65S4: A167-A8.

39. Beers E, Moerkerken DC, Leufkens HG, Egberts TC, Jansen PA. Participation of older people in preauthorization trials of recently approved medicines. J Am Geriatr Soc 2014; 62: 1883-90.

40. Bellingtier JA, Neupert SD. Negative aging attitudes predictgreater reactivity to daily stressors in older adults. J Gerontol B Psychol Sci Soc Sci 2018; 73: 1155-59.

41. Bendick M, Jr., Brown LE, Wall K. No foot in the door: an experimental study of employment discrimination against older workers. J Aging Soc Policy 1999; 10: 5-23.

42. Bendick M, Jr., Jackson CW, Romero JH. Employment discrimination against older workers: an experimental study of hiring practices. J Aging Soc Policy 1996; 8: 25-46.

43. Bennett CL, Greenfield S, Aronow H, Ganz P, Vogelzang NJ, Elashoff RM. Patterns of care related to age of men with prostate cancer. Cancer 1991; 67: 2633-41.

44. Benraad CE, Kamerman-Celie F, van Munster BC, Oude Voshaar RC, Spijker J, Olde Rikkert MG. Geriatric characteristics in randomised controlled trials on antidepressant drugs for older adults: a systematic review. Int J Geriatr Psychiatry 2016; 31: 990-1003.

45. Bensadon BA. Memory self-efficacy and stereotype effects in aging. PhD thesis, University of Florida, 2011.

46. Bergman L, Dekker G, van Kerkhoff EH, Peterse HL, van Dongen JA, van Leeuwen FE. Influence of age and comorbidity on treatment choice and survival in elderly patients with breast cancer. Breast Cancer Res Treat 1991; 18: 189-98.

47. Bergman L, Dekker G, van Leeuwen FE, Huisman SJ, van Dam FS, van Dongen JA. The effect of age on treatment choice and survival in elderly breast cancer patients. Cancer 1991; 67: 2227-34.

48. Bergman YS, Bodner E, Shrira A. Subjective nearness to death and end-of-life anxieties: the moderating role of ageism. Aging Ment Health 2018; 22: 678-85.

49. Bernardes SF, Marques S, Matos M. Old and in pain: enduring and situational effects of cultural aging stereotypes on older people's pain experiences. Eur J Pain 2015; 19: 994-1001.

50. Beyer AK, Wolff JK, Warner LM, Schüz B, Wurm S. The role of physical activity in the relationship between self-perceptions of ageing and self-rated health in older adults. Psychol Health 2015; 30: 671-85.

51. Bibby CL. Perceptions of age discrimination, organizational justice, and employee attitudes on intentions to leave in the engineering industry. PhD thesis, Lynn University, 2000.

52. Bilinska P, Wegge J, Kliegel M. Caring for the elderly but not for one’s own old employees? Organizational age climate, age stereotypes, and turnover intentions in young and old nurses. J Pers Psychol 2016; 15: 95-105.

53. Blackman IC, Bond M, Bowling A, et al. Age and sex do not bias the use of angiotensin-converting enzyme inhibitors in acute myocardial infarction and congestive heart failure. J Am Geriatr Soc 2003; 51: 572-73.

54. Blosser CD, Huverserian A, Bloom RD, et al. Age, exclusion criteria, and generalizability of randomized trials enrolling kidney transplant recipients. Transplantation 2011; 91: 858-63.

55. Bond M, Bowling A, McKee D, et al. Does ageism affect the management of ischaemic heart disease? J Health Serv Res Policy 2003; 8: 40-47.

56. Bouchardy C, Rapiti E, Fioretta G, et al. Undertreatment strongly decreases prognosis of breast cancer in elderly women. J Clin Oncol 2003; 21: 3580-87.

57. Bouman WP, Arcelus J. Are psychiatrists guilty of "ageism" when it comes to taking a sexual history? Int J Geriatr Psychiatry 2001; 16: 27-31.

58. Bowen CE, Skribecc V. Old age expectations are related to how long people want to live. Ageing Soc 2017; 37: 1898-923.

59. Bowen CE, Staudinger UM. Relationship between age and promotion orientation depends on perceived older worker stereotypes. J Gerontol B Psychol Sci Soc Sci 2013; 68: 59-63.

60. Bowling A, Bond M, McKee D, et al. Equity in access to exercise tolerance testing, coronary angiography, and coronary artery bypass grafting by age, sex and clinical indications. Heart 2001; 85: 680-86.

61. Bowling A, Mariotto A, Evans O. Are older people willing to give up their place in the queue for cardiac surgery to a younger person? Age Ageing 2002; 31: 187-92.

62. Boyd K, Teres D, Rapoport J, Lemeshow S. The relationship between age and the use of DNR orders in critical care patients. Evidence for age discrimination. Arch Intern Med 1996; 156: 1821-26.

63. Brothers A, Miche M, Wahl HW, Diehl M. Examination of associations among three distinct subjective aging constructs and their relevance for predicting developmental correlates. J Gerontol B Psychol Sci Soc Sci 2017; 72: 547-60.

64. Brown JS, Eraut D, Trask C, Davison AG. Age and the treatment of lung cancer. Thorax 1996; 51: 564-68.

65. Bryant C, Bei B, Gilson K, Komiti A, Jackson H, Judd F. The relationship between attitudes to aging and physical and mental health in older adults. Int Psychogeriatr 2012; 24: 1674-83.

66. Buckley M, O'Neill D. Ageism in Studies of Rehabilitation in Parkinson's Disease. J Am Geriatr Soc 2015; 63: 1470-71.

67. Bugeja G, Kumar A, Banerjee AK. Exclusion of elderly people from clinical research: a descriptive study of published reports. BMJ 1997; 315: 1059.

68. Bustillos A, Fernández Ballesteros R. Attributions of competence mediate the behaviors of caregivers and older adults. GeroPsych 2013; 26: 211-17.

69. Chandra H, Yarzebski J, Goldberg RJ, et al. Age-related trends (1986-1993) in the use of thrombolytic agents in patients with acute myocardial infarction- the Worcester Heart Attack Study. Arch Intern Med 1997; 157: 741-46.

70. Chapman L, Sargent-Cox K, Horswill MS, Anstey KJ. The impact of age stereotypes on older adults' hazard perception performance and driving confidence. J Appl Gerontol 2016; 35: 642-52.

71. Chen Y, King BE. Intra- and intergenerational communication satisfaction as a function of an individual's age and age stereotypes. Int J Behav Dev 2002; 26: 562-70.

72. Cheng ST. Self-perception of aging and satisfaction with children's support. J Gerontol B Psychol Sci Soc Sci 2017; 72: 782-91.

73. Cheng ST, Yip LC, Jim OT, Hui AN. Self-perception of aging and acute medical events in chronically institutionalized middle-aged and older persons with schizophrenia. Int J Geriatr Psychiatry 2012; 27: 907-13.

74. Cherry KE, Brigman S, Reese-Melancon C, Burton-Chase A, Holland K. Memory aging knowledge and memory self-appraisal in younger and older adults. Educ Gerontol 2013; 39: 168-78.

75. Cherubini A, Oristrell J, Pla X, et al. The persistent exclusion of older patients from ongoing clinical trials regarding heart failure. Arch Intern Med 2011; 171: 550-56.

76. Cheung F, Wu AMS, Yeung DY. Factors associated with work volition among aging workers in Hong Kong. J Career Dev 2016; 43: 160-76.

77. Chiu WCK, Chan AW, Snape E, Redman T. Age stereotypes and discriminatory attitudes toward older workers: An East-West comparison. Hum Relat 2001; 54: 629-61.

78. Chu J, Diehr P, Feigl P, et al. The effect of age on the care of women with breast cancer in community hospitals. J Gerontol 1987; 42: 185-90.

79. Chun H, Kang M, Cho SI, Jung-Choi K, Jang SN, Khang YH. Does the experience of discrimination affect health? A cross-sectional study of Korean elders. Asia Pac J Public Health 2015; 27: NP2285-95.

80. Cleveland JN, Landy FJ. The influence of rater and ratee age on two performance judgements. J Pers Psychol 1981; 34: 19-29.

81. Coudin G, Alexopoulos T. 'Help me! I'm old!' How negative aging stereotypes create dependency among older adults. Aging Ment Health 2010; 14: 516-23.

82. Cruz-Jentoft AJ, Carpena-Ruiz M, Montero-Errasquín B, Sánchez-Castellano C, Sánchez-García E. Exclusion of older adults from ongoing clinical trials about type 2 diabetes mellitus. J Am Geriatr Soc 2013; 61: 734-38.

83. Cruz-Jentoft AJ, Gutierrez B. Upper age limits in studies submitted to a research ethics committee. Aging Clin Exp Res 2010; 22: 175-78.

84. Davison EH. The interrelationships among subjective well-being, gender role flexibility, perceived sexism, and perceived ageism in older women. PhD thesis, University of California, Santa Barbara, 2000.

85. de Gara CJ, Basrur V, Figueredo A, Goodyear M, Knight P. The influence of age on the management of anal cancer. Hepatogastroenterology 1995; 42: 73-76.

86. de Rijke JM, Schouten LJ, Schouten HC, Jager JJ, Koppejan AG, van den Brandt PA. Age-specific differences in the diagnostics and treatment of cancer patients aged 50 years and older in the province of Limburg, The Netherlands. Ann Oncol 1996 ; 7: 677-85.

87. DeMichele A, Putt M, Zhang Y, Glick JH, Norman S. Older age predicts a decline in adjuvant chemotherapy recommendations for patients with breast carcinoma: evidence from a tertiary care cohort of chemotherapy-eligible patients. Cancer 2003; 97: 2150-59.

88. Detwiler BP. Minority stress in the sexual minority older adult population: exploring the relationships among discrimination, mental health, and quality of life. PhD thesis, Lehigh University, 2016.

89. DeWilde S, Carey IM, Bremner SA, Richards N, Hilton SR, Cook DG. Evolution of statin prescribing 1994-2001: a case of agism but not of sexism? Heart 2003 ; 89: 417-21.

90. Dharma-Wardene MW, De Gara C, Au HJ, Hanson J, Hatcher J. Ageism in rectal carcinoma? Treatment and outcome variations. Int J Gastrointest Cancer 2002; 32: 129-38.

91. Di Rosa M, Chiatti C, Rimland JM, et al. Ageism and surgical treatment of breast cancer in Italian hospitals. Aging Clin Exp Res. 2018; 30: 139-44.

92. Dodd KS, Saczynski JS, Zhao Y, Goldberg RJ, Gurwitz JH. Exclusion of older adults and women from recent trials of acute coronary syndromes. J Am Geriatr Soc 2011; 59: 506-11.

93. Dordoni P, Van der Heijden B, Peters P, Kraus-Hoogeveen S, Argentero P. Keep up the good work! Age-moderated mediation model on intention to retire. Front Psychol 2017; 8: 1717.

94. Drydakis N, MacDonald P, Chiotis V, Somers L. Age discrimination in the UK labour market. Does race moderate ageism? An experimental investigation. Appl Econ Lett 2018 ; 25: 1-4.

95. Du X, Goodwin JS. Increase of chemotherapy use in older women with breast carcinoma from 1991 to 1996. Cancer 2001; 92: 730-37.

96. Dudley NJ, Bowling A, Bond M, et al. Age- and sex-related bias in the management of heart disease in a district general hospital. Age Ageing 2002; 31: 37-42.

97. Dudley NJ, Burns E. Influence of age on policies for admission and thrombolysis in coronary care units in the United Kingdom. Age Ageing 1992; 21: 95-98.

98. Earle CC, Venditti LN, Neumann PJ, et al. Who gets chemotherapy for metastatic lung cancer? Chest 2000; 117: 1239-46.

99. El Bcheraoui C, Adib S, Chapuis-Lucciani N. Perception of ageism and self-esteem among Lebanese elders at home and abroad. J Med Liban 2015; 63: 27-33.

100. Elder AT, Shaw TRD, Turnbull CM, Starkey IR. Elderly and younger patients selected to undergo coronary angiography. BMJ 1991; 303: 950-53.

101. Emile M, Chalabaev A, Colson SS, Vaulerin J, Falzon C, D'Arripe-Longueville F. Effects of implicit theories of ability and stereotype-inconsistent information on handgrip strength in older adults: A regulatory fit perspective. Psychophysiology 2017; 54: 483-89.

102. Emile M, d'Arripe-Longueville F, Cheval B, Amato M, Chalabaev A. An ego depletion account of aging stereotypes' effects on health-related variables. J Gerontol B Psychol Sci Soc Sci 2015; 70: 876-85.

103. Emile Ml, Chalabaev A, Stephan Y, Corrion K, d'Arripe-Longueville F. Aging stereotypes and active lifestyle: Personal correlates of stereotype internalization and relationships with level of physical activity among older adults. Psychol Sport Exerc 2014; 15: 198-204.

104. Estrella JD, Sánchez-Jurado PM, Abizanda P, et al. Age discrimination in oncological treatments in elderly patients with colon and breast cancer. A case-control study. Revista Espanola de Geriatria y Gerontologia 2005; 40: 78-84.

105. Fairhead JF, Rothwell PM. Underinvestigation and undertreatment of carotid disease in elderly patients with transient ischaemic attack and stroke: comparative population based study. BMJ 2006; 333: 525-27.

106. Fasbender U, Wang M. Negative attitudes toward older workers and hiring decisions: testing the moderating role of decision makers' core self-evaluations. Front Psychol. 2017; 7: 2057.

107. Faure A, Ndobo A. On gender-based and age-based discrimination: when the social ingraining and acceptability of non discriminatory norms matter. Revue Internationale de Psychologie Sociale 2015; 28: 7-43.

108. Fetting JH, Comstock GW, Eby S, et al. The effect of aging on the utilization of chemotherapy for metastatic breast cancer: a population-based study. Cancer Invest 1997; 15: 199-203.

109. Finkelstein LM, Burke MJ. Age stereotyping at work: the role of rater and contextual factors on evaluations of job applicants. J Gen Psychol 1998; 125: 317-45.

110. Fitzsimmons PR, Blayney S, Mina-Corkill S, Scott GO. Older participants are frequently excluded from Parkinson's disease research. Parkinsonism Relat Disord 2012; 18: 585-89.

111. Fitzsimmons PR, Kalaher H, Cooper H, Hussain L, Menezes B. Ageism in stroke research. Int J Stroke 2012; 7: 35-36.

112. Fleming C, D'Agostino RB, Selker HP. Is coronary-care-unit admission restricted for elderly patients? A multicenter study. Am J Public Health 1991; 81: 1121-26.

113. Forman DE, Cannon CP, Hernandez AF, Liang L, Yancy C, Fonarow GC. Influence of age on the management of heart failure: findings from Get With the Guidelines-Heart Failure (GWTG-HF). Am Heart J 2009; 157: 1010-17.

114. Freeman AT, Santini ZI, Tyrovolas S, Rummel-Kluge C, Haro JM, Koyanagi A. Negative perceptions of ageing predict the onset and persistence of depression and anxiety: findings from a prospective analysis of the Irish Longitudinal Study on Ageing (TILDA). J Affect Disord 2016; 199: 132-38.

115. Fritzsche B, Marcus J. The senior discount: biases against older career changers. J Appl Soc Psychol 2013; 43: 350-62.

116. Gaillard M, Desmette D. (In)validating stereotypes about older workers influences their intentions to retire early and to learn and develop. Basic Appl Soc Psych 2010; 32: 86-98.

117. Garstka TA, Schmitt MT, Branscombe NR, Hummert ML. How young and older adults differ in their responses to perceived age discrimination. Psychol Aging 2004; 19: 326-35.

118. Gaynor EJ, Geoghegan SE, O'Neill D. Ageism in stroke rehabilitation studies. Age Ageing 2014; 43: 429-31.

119. Gewirtz-Meydan A, Ayalon L. Physicians' response to sexual dysfunction presented by a younger vs. an older adult. Int J Geriatr Psychiatry 2017; 32: 1476-83.

120. Gibney S, Moran N, Ward M, Shannon S. The effects of discrimination and perceptions of ageism on community participation among older adults. Age Ageing 2017; 46S3:1-2.

121. Ginsburg AB, Goldstein SG. Age bias in referral for psychological consultation. J Gerontol 1974; 29: 410-15.

122. Giron MS, Fastbom J, Winblad B. Clinical trials of potential antidepressants: to what extent are the elderly represented: a review. Int J Geriatr Psychiatry 2005; 20: 201-17.

123. Giugliano RP, Camargo CA, Jr., Lloyd-Jones DM, et al. Elderly patients receive less aggressive medical and invasive management of unstable angina: potential impact of practice guidelines. Arch Intern Med 1998; 158: 1113-20.

124. Gnavi R, Migliardi A, Demaria M, Petrelli A, Caprioglio A, Costa G. Statins prescribing for the secondary prevention of ischaemic heart disease in Torino, Italy. A case of ageism and social inequalities. Eur J Public Health 2007; 1: 492-96.

125. Goodwin JS, Hunt WC, Samet JM. Determinants of cancer therapy in elderly patients. Cancer 1993; 72: 594-601.

126. Gordon RA, Rozelle RM, Baxter JC. The effect of applicant age, job level, and accountability on the evaluation of job applicants. Organ Behav Hum Decis Process 1988; 41: 20-33.

127. Gosney M. Under-investigation of older people with abnormal chest radiographs. Gerontology 2005; 51: 1-6.

128. Goyal P, Sterling MR, Beecy AN, et al. Patterns of scheduled follow-up appointments following hospitalization for heart failure: insights from an urban medical center in the United States. Clin Interv Aging 2016; 11: 1325-32.

129. Graf AS, Patrick JH. The influence of sexual attitudes on mid- to late-life sexual well-being: Age, not gender, as a salient factor. Int J Aging Hum Dev 2014; 79: 55-79.

130. Grant PT, Henry JM, McNaughton GW. The management of elderly blunt trauma victims in Scotland: Evidence of ageism? Injury 2000; 31: 519-28.

131. Green P, Maurer MS, Foody JM, Forman DE, Wenger NK. Representation of older adults in the late-breaking clinical trials American Heart Association 2011 Scientific Sessions. J Am Coll Cardiol 2012; 60: 869-71.

132. Greene MG. Ageism in the medical encounter: an exploratory study of the physician-elderly patient relationship. Lang Commun 1984: 83.

133. Greenfield S, Blanco DM, Elashoff RM, Ganz PA. Patterns of care related to age of breast cancer patients. JAMA 1987; 257: 2766-70.

134. Gringart E, Helmes E. Age discrimination in hiring practices against older adults in Western Australia: the case of accounting assistants. Australas J Ageing 2001; 20: 23-28.

135. Gu D, Dupre ME, Qiu L. Self-perception of uselessness and mortality among older adults in China. Arch Gerontol Geriatr 2017; 68: 186-94.

136. Guadagnoli E, Shapiro C, Gurwitz JH, et al. Age-related patterns of care: evidence against ageism in the treatment of early-stage breast cancer. J Clin Oncol 1997 ; 15: 2338-44.

137. Gum AM, Ayalon L. Self-perceptions of aging mediate the longitudinal relationship of hopelessness and depressive symptoms. Int J Geriatr Psychiatry 2018; 33: 591-97.

138. Gupta P, Shekhar R, O'Mahony MS. Ageism and sexism in clinical research: are we making any progress? Age Ageing 2013; 42S3: 12.

139. Gurwitz JH, Col NF, Avorn J. Exclusion of the elderly and women from clinical trials in acute myocardial infarction. JAMA 1992; 268: 1417-22.

140. Gurwitz JH, Goldberg RJ, Malmgren JA, et al. Hospital transfer of patients with acute myocardial infarction: the effects of age, race, and insurance type. Am J Med 2002; 112: 528-34.

141. Gurwitz JH, Gore JM, Goldberg RJ, Rubison M, Chandra N, Rogers WJ. Recent age-related trends in the use of thrombolytic therapy in patients who have had acute myocardial infarction. National registry of myocardial infarction. Ann Intern Med 1996; 124: 283-91.

142. Gurwitz JH, Osganian V, Goldberg RJ, Chen ZY, Gore JM, Alpert JS. Diagnostic testing in acute myocardial infarction: does patient age influence utilization patterns? The Worcester heart attack study. Am J Epidemiol 1991; 134: 948-57.

143. Hadbavna A, Coughlan T, Collins DR, O'Neill D. Are we ageist when it comes to interventional stroke studies? Age Ageing 2013; 42S2: 31-32.

144. Haden AR, Butler J. Length of resuscitation attempt versus age-an ageist approach? Age Ageing 2012; 42S2:71.

145. Hajjar I, Miller K, Hirth V. Age-related bias in the management of hypertension: a national survey of physicians' opinions on hypertension in elderly adults. J Gerontol A Biol Sci Med Sci 2002; 57: M487-91.

146. Hall P, Handforth C, Spencer K, Jackson D, Hall G. Age as an independent predictor of chemotherapy treatment decisions in 20 common cancers. J Clin Oncol 2014; 15: 9550.

147. Hamaker ME, Stauder R, van Munster BC. Exclusion of older patients from ongoing clinical trials for hematological malignancies: an evaluation of the National Institutes of Health Clinical Trial Registry. Oncologist 2014; 19: 1069-75.

148. Hamel MB, Teno JM, Goldman L, et al. Patient age and decisions to withhold life-sustaining treatments from seriously ill, hospitalized adults. SUPPORT Investigators. Study to understand prognoses and preferences for outcomes and risks of treatment. Ann Intern Med 1999; 130: 116-25.

149. Han J. The roles of self and society in the relationship between physical health, self-perception of aging, and depressive symptoms in later life. PhD thesis, The Ohio State University, 2012.

150. Han J. Chronic illnesses and depressive symptoms among older people: functional limitations as a mediator and self-perceptions of aging as a moderator. J Aging Health 2017; 30: 1118-1204.

151. Han J, Richardson VE. The relationships among perceived discrimination, self-perceptions of aging, and depressive symptoms: a longitudinal examination of age discrimination. Aging Ment Health 2015; 19: 747-55.

152. Harries C, Forrest D, Harvey N, McClelland A, Bowling A. Which doctors are influenced by a patient's age? A multi-method study of angina treatment in general practice, cardiology and gerontology. Qual Saf Health Care 2007; 16: 23-7.

153. Harrison MJ, Kim CA, Silverberg M, Paget SA. Does age bias the aggressive treatment of elderly patients with rheumatoid arthritis? J Rheumatol 2005 ; 32: 1243-48.

154. Hassell BL, Perrewé PL. An examination of the relationship between older workers' perceptions of age discrimination and employee psychological states. J Managerial Issues 1993; 5: 109-20.

155. Hausdorff JM, Levy BR, Wei JY. The power of ageism on physical function of older persons: reversibility of age-related gait changes. J Am Geriatr Soc 1999; 47: 1346-49.

156. Heal HC, Husband HJ. Disclosing a diagnosis of dementia: is age a factor? Aging Ment Health 1998; 2: 144-50.

157. Hehman JA. Costs and potential mitigating factors of the stigmatization of older adults. PhD thesis, University of California, Santa Barbara, 2010.

158. Hehman JA, Bugental DB. Responses to patronizing communication and factors that attenuate those responses. Psychol Aging 2015; 30: 552-60.

159. Heiat A, Gross CP, Krumholz HM. Representation of the elderly, women, and minorities in heart failure clinical trials. Arch Intern Med 2002; 162: 1682-88.

160. Helmes E, Gee S. Attitudes of Australian therapists toward older clients: educational and training imperatives. Educ Gerontol 2003 ; 29: 657-70.

161. Herman RE, Williams KN. Elderspeak's influence on resistiveness to care: focus on behavioral events. Am J Alzheimers Dis Other Demen 2009; 24: 417-23.

162. Hertzman J, Zhong Y. A model of hospitality students' attitude toward and willingness to work with older adults. Int J Contemp Hosp Manag 2016; 28: 681-99.

163. Hillson SD, Rich EC, Dowd BE, Luxenberg MG. Impact of intern workload on length of hospital stay for elderly patients. Gerontol Geriatr Educ 1993; 14: 33-41.

164. Hofstetter H, Cohen A. The mediating role of job content plateau on the relationship between work experience characteristics and early retirement and turnover intentions. Pers Rev 2014; 43: 350-76.

165. Hori A, Shibata T, Kami M, et al. Age disparity between a cancer population and participants in clinical trials submitted as a new drug application of anticancer drugs in Japan. Cancer 2007; 109: 2541-46.

166. Horton S, Baker J, Pearce W, Deakin JM. Immunity to popular stereotypes of aging? Seniors and stereotype threat. Educ Gerontol 2010; 36: 353-71.

167. Hou Y, Zhang D, Gu J, et al. The association between self-perceptions of aging and antihypertensive medication adherence in older Chinese adults. Aging Clin Exp Res 2016; 28: 1113-20.

168. Hughes ML. The influence of self-perceptions of aging on older adults' cognition and behavior. PhD thesis, Texas A&M University, 2016.

169. Hummert ML, Shaner JL, Garstka TA, Henry C. Communication with older adults: the influence of age stereotypes, context, and communicator age. Hum Commun Res 1998; 25: 124-51.

170. Humphreys K, Weingardt KR, Horst D, Joshi AA, Finney JW. Prevalence and predictors of research participant eligibility criteria in alcohol treatment outcome studies, 1970-98. Addiction 2005; 100: 1249-57.

171. Hurria A, Leung D, Trainor K, Borgen P, Norton L, Hudis C. Factors influencing treatment patterns of breast cancer patients age 75 and older. Crit Rev Oncol Hematol 2003; 46: 121-26.

172. Hutchens RM. Do job opportunities decline with age? ILR Review 1988 ; 42: 89-99.

173. Hutchins LF, Unger JM, Crowley JJ, Coltman CA, Jr., Albain KS. Underrepresentation of patients 65 years of age or older in cancer-treatment trials. N Engl J Med 1999 ; 341: 2061-67.

174. Huy C, Schneider S, Thiel A. Perceptions of aging and health behavior: determinants of a healthy diet in an older German population. J Nutr Health Aging 2010 14: 381-85.

175. Iecovich E, Avivi M. Agism and burnout among nurses in long-term care facilities in Israel. Aging Ment Health 2017; 21: 327-35.

176. Jazieh AR, Buncher CR. Racial and age-related disparities in obtaining screening mammography: results of a statewide database. South Med J 2002; 95: 1145-48.

177. Jennens RR, Giles GG, Fox RM. Increasing underrepresentation of elderly patients with advanced colorectal or non-small-cell lung cancer in chemotherapy trials. Intern Med J 2006; 36: 216-20.

178. Jerant AF, Franks P, Jackson JE, Doescher MP. Age-related disparities in cancer screening: analysis of 2001 Behavioral Risk Factor Surveillance System data. Ann Fam Med 2004; 2: 481-87.

179. Joanisse M, Gagnon S, Voloaca M. The impact of stereotype threat on the simulated driving performance of older drivers. Accid Anal Prev 2013; 50: 530-38.

180. Johnson MF, Kramer AM. Physicians' responses to clinical scenarios involving life-threatening illness vary by patients' age. J Clin Ethic 2000; 11: 323-27.

181. Johnson RW, Neumark D. Age discrimination, job separations, and employment status of older workers: evidence from self-reports. J Hum Resour 1997; 32: 779-811.

182. Kager MB. Factors that affect hiring: a study of age discrimination and hiring. PhD thesis, University of Massachusetts, Boston, 2000.

183. Kahana EF, Kiyak HA. Attitudes and behavior of staff in facilities for the aged. Res Aging 1984; 6: 395-416.

184. Kandel R, Benerjee J, Thakur S, et al. Functional status, comorbidities and their impact on management of older patients with cancer in India. Eur Geriatr Med 2014; S75.

185. Karpinska K, Henkens K, Schippers J. The recruitment of early retirees: a vignette study of the factors that affect managers' decisions. Ageing Soc 2011; 31: 570-89.

186. Karpinska K, Henkens K, Schippers J. Retention of older workers: Impact of managers' age norms and stereotypes. Eur Sociol Rev 2013; 18: 1312-35.

187. Kaufmann MC, Krings F, Sczesny S. Looking too old? How an older age appearance reduces chances of being hired. Br J Manag 2016; 27: 727-39.

188. Kawai N, Kubo K, Kubo-Kawai N. 'Granny dumping': Acceptability of sacrificing the elderly in a simulated moral dilemma. Jpn Psychol Res 2014; 56: 254-62.

189. Kessler EM, Schneider T. Do treatment attitudes and decisions of psychotherapists-in-training depend on a patient's age? J Gerontol B Psychol Sci Soc Sci 2017; published online June 13. DOI: 10.1093/geronb/gbx078.

190. Kim ES, Moored KD, Giasson HL, Smith J. Satisfaction with aging and use of preventive health services. Prev Med 2014; 69: 176-80.

191. Kim G, Jang Y, Chiriboga DA. Personal views about aging among Korean American older adults: the role of physical health, social network, and acculturation. J Cross Cult Gerontol 2012; 27: 139-48.

192. Kim H. Older workers and their selection of partial and full retirement: evidence from Health and Retirement Study . PhD thesis, Pursue University, 2002.

193. Kim H. The mechanism of ageism: The relationship between perceived ageism and depressive symptoms in later life. Rejuvenation Res 2016; 19: 97-8.

194. Kim I-H, Noh S, Chun H. Mediating and moderating effects in ageism and depression among the Korean elderly: the roles of emotional reactions and coping reponses. Osong Public Health Res Perspect 2016; 7: 3-11.

195. Kim SH. Older people's expectations regarding ageing, health-promoting behaviour and health status. J Adv Nurs 2009; 65: 84-91.

196. Kirchner C, Völker I, Bock OL. Priming with age stereotypes influences the performance of elderly workers. Psychol 2015; 6: 133-37.

197. Kleindorfer D, Meganathan K, Adeoye O, Khatri P, Hornung R. Elderly ischemic stroke patients are less likely to be treated with rt-pa. Stroke 2012; 43S1: 3141

198. Kluge A, Krings F. Attitudes toward the older workers and human resources. Swiss J Psychol 2008; 67: 61-64.

199. Klusmann V, Sproesser G, Wolff JK, Renner B, Neupert S. Positive self-perceptions of aging promote healthy eating behavior across the life span via social-cognitive processes. J Gerontol B Psychol Sci Soc Sci 2017; published online November 28. DOI: 10.1093/geronb/gbx139.

200. Kokoszka A, Kot W. Relationship of the patient age and intensity of type 2 diabetes treatment. Pol Arch Med Wewn 2007 Sep; 117: 396-401.

201. Konrat C, Boutron I, Trinquart L, Auleley GR, Ricordeau P, Ravaud P. Underrepresentation of elderly people in randomised controlled trials. The example of trials of 4 widely prescribed drugs. PLoS One 2012; 7: e33559.

202. Kotter-Grühn D, Kleinspehn-Ammerlahn A, Gerstorf D, Smith J. Self-perceptions of aging predict mortality and change with approaching death: 16-Year longitudinal results from the Berlin aging study. Psychol Aging 2009; 24: 654-67.

203. Krings F, Szczerbinska K, Kluge A. Stereotypical inference as mediators of age discrimination: the role of competence and warmth. Br J Manag 2010; 22: 187-201.

204. Krumholz HM, Friesinger GC, Cook EF, Lee TH, Rouan GW, Goldman L. Relationship of age with eligibility for thrombolytic therapy and mortality among patients with suspected acute myocardial infarction. J Am Geriatr Soc 1994; 42: 127-31.

205. Kucharski LT, White RM, Jr., Schratz M. Age bias, referral for psychological assistance and the private physician. J Gerontol 1979; 34: 423-28.

206. Kvitek SDB, Shaver BJ, Blood H, Shepard KF. Age bias: physical therapists and older patients. J Gerontol. 1986; 41: 706-09.

207. Kwak M, Ingersoll-Dayton B, Burgard S. Receipt of care and depressive symptoms in later life: the importance of self-perceptions of aging. J Gerontol B Psychol Sci Soc Sci 2014; 69: 325-35.

208. Lagace M, Charmarkeh H, Laplante J, Tanguay A. How ageism contributes to the second-level digital divide: the case of Canadian seniors. J Technol Hum Usability 2015; 11: 1-13.

209. Lagacé M, Tougas F, Laplante J, Neveu JF. Health in danger: Repercussions of the ageist on psychological disengagement and self-esteem of nurses 45 years and older. Can J Aging 2008; 27: 285-99.

210. Lahey J. Age, women and hiring: an experimental study. J Hum Resour 2006; 43: 30-56

211. Lai DW. Older chinese' attitudes toward aging and the relationship to mental health: an international comparison. Soc Work Health Care 2009; 48: 243-59.

212. Lain D. Working past 65 in the UK and the USA: segregation into ‘Lopaq’ occupations? Work Employ Soc 2012; 26: 78-94.

213. Lambert AE, Watson JM, Stefanucci JK, Ward N, Bakdash JZ, Strayer DL. Stereotype threat impairs older adult driving. Appl Cogn Psychol 2016; 30: 22-28.

214. Lavelle K, Moran A, Howell A, Bundred N, Campbell M, Todd C. Older women with operable breast cancer are less likely to have surgery. Br J Surg 2007; 94: 1209-15.

215. Lavelle K, Sowerbutts AM, Bundred N, et al. Is lack of surgery for older breast cancer patients in the UK explained by patient choice or poor health? A prospective cohort study. Br J Cancer 2014; 110: 573-83.

216. Lavelle K, Todd C, Moran A, Howell A, Bundred N, Campbell M. Non-standard management of breast cancer increases with age in the UK: a population based cohort of women > or =65 years. Br J Cancer 2007; 96: 1197-203.

217. Lazazzara A, Karpinksa K, Henkens K. What factors influence training opportunities for older workers? Three factorial surveys exploring the attitudes of HR professionals. Int J Hum Resour Manage 2013; 24: 2154-72.

218. Lee HS, Kim C. Structural equation modeling to assess discrimination, stress, social support, and depression among the elderly women in South Korea. Asian Nurs Res 2016; 10: 182-88.

219. Lee HS, Kim GH, Jung SW, Lee JH, Lee KJ, Kim JJ. The association between perceived discriminations and well-being in Korean employed workers: The 4th Korean working conditions survey. Ann Occup Environ Med 2017; 29: 46.

220. Lee PY, Alexander KP, Hammill BG, Pasquali SK, Peterson ED. Representation of elderly persons and women in published randomized trials of acute coronary syndromes. JAMA 2001; 286: 708-13.

221. Lehmann R, Beekley A, Casey L, Salim A, Martin M. The impact of advanced age on trauma triage decisions and outcomes: a statewide analysis. Am J Surg 2009; 197: 571-75.

222. Leinonen A, Koponen M, Hartikainen S. Systematic review: representativeness of participants in RCTs of Acetylcholinesterase Inhibitors. PLoS One 2015; 10: e0124500.

223. Leinsink PLM, Knies E. Line managers' support for older workers. Int J Hum Resour Manage 2011; 22: 1902-17.

224. Levy BR. Handwriting as a reflection of aging self-stereotypes. J Geriatr Psychiatry 2000; 33: 81-94.

225. Levy BR, Ashman O, Dror I. To be or not to be: the effects of aging stereotypes on the will to live. Omega(Westport) 1999; 40: 409-20.

226. Levy BR, Kosteas J, Slade M, Myers L. Exclusion of elderly persons from health-risk behavior clinical trials. Prev Med 2006; 43: 80-85.

227. Levy BR, Bavishi A. Survival advantage mechanism: inflammation as a mediator of positive self-perceptions of aging on longevity. J Gerontol B Psychol Sci Soc Sci 2018; 73: 409-12.

228. Levy BR, Ding L, Lakra D, Kosteas J, Niccolai L. Older persons' exclusion from sexually transmitted disease risk-reduction clinical trials. Sex Transm Dis 2007; 34: 541-44.

229. Levy BR, Slade MD, May J, Caracciolo EA. Physical recovery after acute myocardial infarction: positive age self-stereotypes as a resource. Int J Aging Hum Dev 2006; 62: 285-301.

230. Levy BR, Ferrucci L, Zonderman AB, Slade MD, Troncoso J, Resnick SM. A culture-brain link: negative age stereotypes predict Alzheimer's disease biomarkers. Psychol Aging 2016; 31: 82-88.

231. Levy BR, Hausdorff JM, Hencke R, Wei JY. Reducing cardiovascular stress with positive self-stereotypes of aging. J Gerontol B Psychol Sci Soc Sci 2000; 55: P205-13.

232. Levy BR, Leifheit-Limson E. The stereotype-matching Effect: greater influence on functioning when age stereotypes correspond to outcomes. Psychol Aging 2009; 24: 230-33.

233. Levy BR, Moffat S, Resnick SM, Slade MD, Ferrucci L. Buffer against cumulative stress positive age self-stereotypes predict lower cortisol across 30 Years. GeroPsych (Bern) 2016; 29: 141-46.

234. Levy BR, Myers LM. Preventive health behaviors influenced by self-perceptions of aging. Prev Med 2004; 39: 625-9.

235. Levy BR, Myers LM. Relationship between respiratory mortality and self-perceptions of aging. Psychol Health 2005; 20: 553-64.

236. Levy BR, Pilver C, Chung PH, Slade MD. Subliminal strengthening: improving older individuals' physical function over time with an implicit-age-stereotype intervention. Psychol Sci 2014; 25: 2127-35.

237. Levy BR, Pilver CE, Pietrzak RH. Lower prevalence of psychiatric conditions when negative age stereotypes are resisted. Soc Sci Med 2014; 119: 170-74.

238. Levy BR, Ryall AL, Pilver CE, Sheridan PL, Wei JY, Hausdorff JM. Influence of African American elders' age stereotypes on their cardiovascular response to stress. Anxiety Stress Coping 2008; 21: 85-93.

239. Levy BR, Schlesinger MJ. When self-interest and age stereotypes collide: elders opposing increased funds for programs benefiting themselves. J Aging Soc Policy 2005; 17: 25-39.

240. Levy BR, Slade MD, Chung PH, Gill TM. Resiliency over time of elders' age stereotypes after encountering stressful events. J Gerontol B Psychol Sci Soc Sci 2015 ; 70: 886-90.

241. Levy BR, Slade MD, Gill TM. Hearing decline predicted by elders' stereotypes. J Gerontol B Psychol Sci Soc Sci 2006; 61: P82-87.

242. Levy BR, Slade MD, Kasl SV. Longitudinal benefit of positive self-perceptions of aging on functional health. J Gerontol B Psychol Sci Soc Sci 2002; 57: P409-17.

243. Levy BR, Slade MD, Kunkel SR, Kasl SV. Longevity increased by positive self-perceptions of aging. J Pers Soc Psychol 2002; 83: 261-70.

244. Levy BR, Slade MD, Murphy TE, Gill TM. Association between positive age stereotypes and recovery from disability in older persons. JAMA 2012; 308: 1972-73.

245. Levy BR, Zonderman AB, Slade MD, Ferrucci L. Age stereotypes held earlier in life predict cardiovascular events in later life. Psychol Sci 2009; 20: 296-98.

246. Levy BR, Zonderman AB, Slade MD, Ferrucci L. Memory shaped by age stereotypes over time. J Gerontol B Psychol Sci Soc Sci 2012; 67: 432-36.

247. Lewis JH, Kilgore ML, Goldman DP, et al. Participation of patients 65 years of age or older in cancer clinical trials. J Clin Oncol 2003; 21: 1383-89.

248. Li X, Lv Q, Li C, Zhang H, Li C, Jin J. The relationship between expectation regarding aging and functional health status among older adults in China. J Nurs Scholarsh 2013; 45: 328-35.

249. Liberopoulos G, Trikalinos NA, Ioannidis JP. The elderly were under-represented in osteoarthritis clinical trials. J Clin Epidemiol 2009; 62: 1218-23.

250. Linden M, Kurtz G. A randomised controlled experimental study on the influence of patient age on medical decisions in respect to the diagnosis and treatment of depression in the elderly. Curr Gerontol Geriatr Res 2009; 1–4.

251. Locher JL, Burgio KL, Goode PS, Roth DL, Rodriguez E. Effects of age and causal attribution to aging on health-related behaviors associated with urinary incontinence in older women. Gerontologist 2002; 42: 515-21.

252. Low G, Molzahn AE, Schopflocher D. Attitudes to aging mediate the relationship between older peoples' subjective health and quality of life in 20 countries. Health Qual Life Outcome 2013; 11: 146.

253. Lu L. Attitudes toward older people and coworkers' intention to work with older employees: a Taiwanese study. Int J Aging Hum Dev 2010; 71: 305-22.

254. Lu L, Kao S, Hsieh Y. Positive attitudes toward older people and well-being among Chinese community older adults. J Appl Gerontol 2010 ; 29: 622-39.

255. Ludbrook JJ, Truong PT, MacNeil MV, et al. Do age and comorbidity impact treatment allocation and outcomes in limited stage small-cell lung cancer? a community-based population analysis. Int J Radiat Oncol Biol Phys 2003; 55: 1321-30.

256. Lund T, Villadsen E. Who retires early and why? Determinants of early retirement pension among Danish employees 57-62 years. Eur J Ageing 2005; 2: 275-80.

257. Lyons A, Alba B, Heywood W, et al. Experiences of ageism and the mental health of older adults. Aging Ment Health 2017; published online August 10. DOI: 10.1080/13607863.2017.1364347.

258. Macdonald JL, Levy SR. Ageism in the workplace: the role of psychosocial factors in predicting job satisfaction, commitment, and engagement. J Soc Issues 2016; 72: 169-90.

259. Mackay JH, Powell SJ, Charman SC, Rozario C. Resuscitation after cardiac surgery: are we ageist? Eur J Anaesthesiol 2004; 21: 66-71.

260. MacNeil RD. Attitudes toward the aged and identified employment preferences of therapeutic recreation students. Educ Gerontol 1991; 17: 543-58.

261. Madan AK, Aliabadi-Wahle S, Beech DJ. Ageism in medical students' treatment recommendations: the example of breast-conserving procedures. Acad Med 2001; 76: 282-4.

262. Madan AK, Aliabadi-Wahle S, Beech DJ. Age bias: a cause of underutilization of breast conservation treatment. J Cancer Educ 2001; 16: 29-32.

263. Madan AK, Cooper L, Gratzer A, Beech DJ. Ageism in breast cancer surgical options by medical students. Tenn Med 2006; 99: 37-41.

264. Mahoney T, Kuo YH, Topilow A, Davis JM. Stage III colon cancers: why adjuvant chemotherapy is not offered to elderly patients. Arch Surg 2000; 135: 182-85.

265. Maier H, Smith J. Psychological predictors of mortality in old age. J Gerontol B Psychol Sci Soc Sci 1999; 54: P44-54.

266. Majeed A, Moser K, Maxwell R. Age, sex and practice variations in the use of statins in general practice in England and Wales. J Public Health Med 2000; 22: 275-9.

267. Malik MK, Tartter PI, Belfer R. Undertreated breast cancer in the elderly. J Cancer Epidemiol 2013.

268. Mandelblatt JS, Hadley J, Kerner JF, et al. Patterns of breast carcinoma treatment in older women: patient preference and clinical and physical influences. Cancer 2000; 89: 561-73.

269. Marchiondo LA, Gonzales E, Williams LJ. Trajectories of perceived workplace age discrimination and long-term associations with mental, self-rated, and occupational health. J Gerontol B Psychol Sci Soc Sci 2017; published online July 12. DOI: 10.1093/geronb/gbx095.

270. Mariotto A, De Leo D, Buono MD, Favaretti C, Austin P, Naylor CD. Will elderly patients stand aside for younger patients in the queue for cardiac services? Lancet 1999; 354: 467-70.

271. Marques S, Lima ML, Abrams D, Swift H. Will to live in older people's medical decisions: immediate and delayed effects of aging stereotypes. J Appl Soc Psychol 2014; 44: 399-408.

272. Marsh S, Johansen A. Timetrends in the age of people included in osteoporosis drug trials. Osteoporosis Int 2016; 27: S674.

273. McLaughlin TJ, Gurwitz JH, Willison DJ, Gao X, Soumerai SB. Delayed thrombolytic treatment of older patients with acute myocardial infarction. J Am Geriatr Soc 1999; 47: 1222-28.

274. McLaughlin TJ, Soumerai SB, Willison DJ, et al. Adherence to national guidelines for drug treatment of suspected acute myocardial infarction: evidence for undertreatment in women and the elderly. Arch Intern Med 1996; 156: 799-805.

275. Meisner BA, Baker J. An exploratory analysis of aging expectations and health care behavior among aging adults. Psychol Aging 2013; 28: 99-104.

276. Mejia ST, Gonzalez R. Couples' shared beliefs about aging and implications for future functional limitations. Gerontologist 2017; 57S2: S149-59.

277. Menkin JA. Social engagement in assisted and independent living residences: role of perceptions of aging and links to health. PhD thesis, University of California, Los Angeles, 2018.

278. Menkin JA, Robles TF, Gruenewald TL, Tanner EK, Seeman TE. Positive expectations regarding aging linked to more new friends in later life. J Gerontol B Psychol Sci Soc Sci 2017; 72: 771-81.

279. Miller SS, Gordon AR, Olsson MJ, Lundström JN, Dalton P. Mind over age-stereotype activation and olfactory function. Chem Senses 2013; 38: 167-74.

280. Minnotte KL. Perceived discrimination and work-to-life conflict among workers in the United States. Sociol Q 2012; 53: 188-210.

281. Mitchell SL, Sullivan EA, Lipsitz LA. Exclusion of elderly subjects from clinical trials for Parkinson disease. Arch Neurol 1997; 54: 1393-8.

282. Molden J, Maxfield M. The impact of aging stereotypes on dementia worry. Eur J Ageing 2017; 14: 29-37.

283. Moor C, Zimprich D, Schmitt M, Kliegel M. Personality, aging self-perceptions, and subjective health: a mediation model. Int J Aging Hum Dev 2006; 63: 241-57.

284. Mor V, Masterson-Allen S, Goldberg RJ, Cummings FJ, Glicksman AS. Relationship between age at diagnosis and treatments received by cancer patients. J Am Geriatr Soc 1985; 33: 585-89.

285. Morgans AK, Smith MR, O'Malley AJ, Keating NL. Bone density testing among prostate cancer survivors treated with androgen-deprivation therapy. Cancer 2013; 119: 863-70.

286. Morse AN, Labin LC, Young SB, Aronson MP, Gurwitz JH. Exclusion of elderly women from published randomized trials of stress incontinence surgery. Obstet Gynecol 2004; 104: 498-503.

287. Moser C, Spagnoli J, Santos-Eggimann B. Self-perception of aging and vulnerability to adverse outcomes at the age of 65-70 years. J Gerontol B Psychol Sci Soc Sci 2011; 66: 675-80.

288. Munro PT, Smith RD, Parke TR. Effect of patients' age on management of acute intracranial haematoma: prospective national study. BMJ 2002; 325: 1001.

289. Murphy J, Whittaker L, Sharma A, Powell S, Gould D, Bakran A. Sexism and ageism in vascular access - Is it justified? J Vasc Access 2011; 12: 106.

290. Murthy VH, Krumholz HM, Gross CP. Participation in cancer clinical trials: race-, sex-, and age-based disparities. JAMA 2004; 291: 2720-26.

291. Naeim A, Hurria A, Leake B, Maly RC. Do age and ethnicity predict breast cancer treatment received? A cross-sectional urban population based study. Breast cancer treatment: age and ethnicity. Crit Rev Oncol Hematol 2006; 59: 234-42.

292. Naylor CD, Levinton CM, Baigrie RS, Goldman BS. Placing patients in the queue for coronary surgery: do age and work status alter Canadian specialists' decisions? J Gen Intern Med 1992; 7: 492-98.

293. Nesargikar PN, Kaur V, Cocker DM, Lengyel J. Consenting for pelvic nerve injury in colorectal surgery: need to address age and gender bias. Ann R Coll Surg Engl 2010; 92: 391-94.

294. Neumark D, Burn I, Button P. Experimental age discrimination evidence and the Heckman critique. Am Econ Rev 2016; 106: 303-08.

295. Neuner JM, Binkley N, Sparapani RA, Laud PW, Nattinger AB. Bone density testing in older women and its association with patient age. J Am Geriatr Soc 2006; 54: 485-89.

296. Newcomb PA, Carbone PP. Cancer treatment and age: patient perspectives. J Natl Cancer Inst 1993; 85: 1580-84.

297. Newschaffer CJ, Penberthy L, Desch CE, Retchin SM, Whittemore M. The effect of age and comorbidity in the treatment of elderly women with nonmetastatic breast cancer. Arch Intern Med 1996; 156: 85-90.

298. Ng R, Allore HG, Monin JK, Levy BR. Retirement as meaningful: positive retirement stereotypes associated with longevity. J Soc Issues 2016; 72: 69-85.

299. North MS, Fiske ST. Resource scarcity and prescriptive attitudes generate subtle, intergenerational older-worker exclusion. J Soc Issues 2016; 72: 122-45.

300. Nuckton TJ, List ND. Age as a factor in critical care unit admissions. Arch Intern Med 1995; 155: 1087-92.

301. O'Hare AM, Kaufman JS, Covinsky KE, Landefeld CS, McFarland LV, Larson EB. Current guidelines for using angiotensin-converting enzyme inhibitors and angiotensin II-receptor antagonists in chronic kidney disease: is the evidence base relevant to older adults? Ann Intern Med 2009; 150: 717-24.

302. Oka RK, Fortmann SP, Varady AN. Differences in treatment of acute myocardial infarction by sex, age, and other factors (the Stanford Five-City Project). Am J Cardiol 1996; 78: 861-65.

303. O'Shea DM, Dotson VM, Fieo RA. Aging perceptions and self-efficacy mediate the association between personality traits and depressive symptoms in older adults. Int J Geriatr Psychiatry 2017; 32: 1217-25.

304. O'Sullivan E. Ageism in Head and Neck Cancer (HNC): Fact or fiction? Ir J Med Sci 2012; 181: S297-98.

305. Paeck T, Ferreira ML, Sun C, Lin CW, Tiedemann A, Maher CG. Are older adults missing from low back pain clinical trials? A systematic review and meta-analysis. Arthritis Care Res 2014; 66: 1220-26.

306. Paggi ME, Jopp DS. Outcomes of occupational self-efficacy in older workers. Int J Aging Hum Dev 2015; 80: 357-78.

307. Paripati H, Tong W, Karlin NJ, Dueck AC, Ross HJ. Treatment and outcomes of elderly versus younger patients with advanced NSCLC at Mayo Clinic Arizona (MCA). J Clin Oncol 2009; 27: e19047.

308. Peake MD, Thompson S, Lowe D, Pearson MG. Ageism in the management of lung cancer. Age Ageing 2003; 32: 171-77.

309. Perry EL, Kulik CT, Bourhis AC. Moderating effects of personal and contextual factors in age discrimination. J Appl Psychol 1996 ; 81: 628-47.

310. Pietrzak RH, Zhu Y, Slade MD, et al. Association between negative age stereotypes and accelerated cellular aging: Evidence from two cohorts of older adults. J Am Geriatr Soc 2016 ; 64: e228-30.

311. Plaisier BR, Blostein PA, Hurt KJ, Malangoni MA. Withholding/withdrawal of life support in trauma patients: is there an age bias? Am Surg 2002; 68: 159-62.

312. Polito MJ. Stereotype threat in older drivers: The stereotype and the threat. PhD thesis, Brandeis University, 2014.

313. Polverino AM. Examining the roles of identity processing styles and self-perceptions of aging on well-being in later life. PhD thesis, Bowling Green State University, 2010.

314. Potosky AL, Harlan LC, Kaplan RS, Johnson KA, Lynch CF. Age, sex, and racial differences in the use of standard adjuvant therapy for colorectal cancer. J Clin Oncol 2002; 20: 1192-202.

315. Protiere C, Viens P, Rousseau F, Moatti JP. Prescribers' attitudes toward elderly breast cancer patients. Discrimination or empathy? Crit Rev Oncol Hematol 2010; 75: 138-50.

316. Rabl T. Age, discrimination, and achievement motives: A study of German employees. Pers Rev 2010; 39: 448-67.

317. Rabl T, del Carmen Triana M. How German employees of different ages conserve resources: perceived age discrimination and affective organizational commitment. Int J Hum Resour Manage 2013; 24: 3599-612.

318. Rabl T, Kühlmann TM. Work-life balance and demographic change: relationships with age and age discrimination. Zeitschrift für Personalpsychologie 2009; 8: 88-99.

319. Rajapakse A, Rajapakse S, Playfer J. Age bias in clinical trials of Parkinson's disease treatment. J Am Geriatr Soc 2008; 56: 2353-54.

320. Rakowski W, Hickey T. Mortality and the attribution of health problems to aging among older adults. Am J Public Health 1992; 82: 1139-41.

321. Ramirez L, Palacios-Espinosa X. Stereotype about old age, social support, ageing anxietyand evaluations of one's own health. J Soc Issues 2016; 72: 47-68.

322. Ray DC, McKinney KA, Ford CV. Differences in psychologists' ratings of older and younger clients. Gerontologist 1987; 27: 82-86.

323. Redman T, Snape E. The consequences of perceived age discrimination amongst older police officers: is social support a buffer? Br J Manage 2006; 17: 167-75.

324. Regueiro CR, Gill N, Hart A, Crawshaw L, Hentosz T, Shannon RP. Primary angioplasty in acute myocardial infarction: does age or race matter? J Thromb Thrombolysis 2003; 15: 119-23.

325. Reid FD, Cook DG, Whincup PH. Use of statins in the secondary prevention of coronary heart disease: is treatment equitable? Heart 2002; 88: 15-19.

326. Reuber M, Torane P, Mac KC. Do older adults have equitable access to specialist epilepsy services? Epilepsia 2010; 51: 2341-43.

327. Richardson B, Webb J, Webber L, Smith K. Age discrimination in the evaluation of job applicants. J Appl Soc Psychol 2013; 43: 35-44.

328. Robertson DA, Kenny RA. 'I'm too old for that'—The association between negative perceptions of aging and disengagement in later life. Pers Individ Dif 2016; 100: 114-19.

329. Robertson DA, King-Kallimanis BL, Kenny RA. Negative perceptions of aging predict longitudinal decline in cognitive function. Psychol Aging 2016; 31: 71-81.

330. Robertson DA, Savva GM, King-Kallimanis BL, Kenny RA. Negative perceptions of aging and decline in walking speed: a self-fulfilling prophecy. PLoS One 2015; 10: e0123260

331. Rochon PA, Fortin PR, Dear KB, Minaker KL, Chalmers TC. Reporting of age data in clinical trials of arthritis. Deficiencies and solutions. Arch Intern Med 1993; 153: 243-8.

332. Rohan EA, Berkman B, Walker S, Holmes W. Geriatric oncology patient: ageism in social work practice. J Gerontol Soc Work 1994; 23: 201-21.

333. Rosen B, Jerdo TH. The influence of age stereotypes on managerial decisions. J Appl Psychol 1976: 61: 428-32.

334. Rosenthal GE, Fortinsky RH. Differences in the treatment of patients with acute myocardial infarction according to patient age. J Am Geriatr Soc 1994; 42: 826-32.

335. Rudd AG, Hoffman A, Down C, Pearson M, Lowe D. Access to stroke care in England, Wales and Northern Ireland: the effect of age, gender and weekend admission. Age Ageing 2007; 36: 247-55.

336. Rupp DE, Vodanovich SJ, Credé M. Age bias in the workplace: the impact of ageism and causal attributions. J Appl Soc Psychol 2006; 36: 1337-64.

337. Ruppel SE, Jenkins WJ, Griffin JL, Kizer JB. Are they depressed or just old? A study of perceptions about the elderly suffering from depression. N Am J Psychol 2010; 12: 31-42.

338. Sabik NJ. Ageism and body esteem: Associations with psychological well-being among late middle-aged African American and European American women. J Gerontol B Psychol Sci Soc Sci 2015; 70: 189-99.

339. Samet J, Hunt WC, Key C, Humble CG, Goodwin JS. Choice of cancer therapy varies with age of patient. JAMA 1986; 255: 3385-90.

340. Sánchez Palacios C, Trianes Torres MV, Blanca Mena MJ. Negative aging stereotypes and their relation with psychosocial variables in the elderly population. Arch Gerontol Geriatr 2009; 48: 385-90.

341. Sargent-Cox KA, Anstey KJ, Luszcz MA. The relationship between change in self-perceptions of aging and physical functioning in older adults. Psychol Aging 2012; 27: 750-60.

342. Sargent-Cox KA, Anstey KJ, Luszcz MA. Longitudinal change of self-perceptions of aging and mortality. J Gerontol B Psychol Sci Soc Sci 2014; 69: 168-73.

343. Sarkisian CA, Hays RD, Mangione CM. Do older adults expect to age successfully? The association between expectations regarding aging and beliefs regarding healthcare seeking among older adults. J Am Geriatr Soc 2002; 50: 1837-43.

344. Sarkisian CA, Lee-Henderson MH, Mangione CM. Do depressed older adults who attribute depression to "old age" believe it is important to seek care? J Gen Intern Med 2003; 18: 1001-05.

345. Sarkisian CA, Prohaska TR, Wong MD, Hirsch S, Mangione CM. The relationship between expectations for aging and physical activity among older adults. J Gen Intern Med 2005; 20: 911-15.

346. Schmitt E. [Active ageing, decreasing capability, social disadvantage and age stereotypes: a contribution to resilience and vulnerability in older adulthood]. Z Gerontol Geriatr 2004; 37: 280-92.

347. Schoenmaker N, Van Gool WA. The age gap between patients in clinical studies and in the general population: a pitfall for dementia research. Lancet Neurol 2004; 3: 627-30.

348. Schrag D, Gelfand SE, Bach PB, Guillem J, Minsky BD, Begg CB. Who gets adjuvant treatment for stage II and III rectal cancer? Insight from surveillance, epidemiology, and end results--Medicare. J Clin Oncol 2001; 19: 3712-18.

349. Schroyen S, Adam S, Marquet M, et al. Communication of healthcare professionals: is there ageism? Eur J Cancer Care 2018; 27.

350. Schroyen S, Marquet M, Jerusalem G, et al. The link between self-perceptions of aging, cancer view and physical and mental health of older people with cancer: a cross-sectional study. J Geriatr Oncol 2017; 8: 64-68.

351. Schroyen S, Missotten P, Jerusalem G, Gilles C, Adam S. Ageism and caring attitudes among nurses in oncology. Int Psychogeriat 2016; 28: 749-57.

352. Schroyen S, Missotten P, Jerusalem G, Van den Akker M, Buntinx F, Adam S. Association between self-perception of aging, view of cancer and health of older patients in oncology: a one-year longitudinal study. BMC Cancer 2017; 17: 614.

353. Seidler AL, Wolff JK. Bidirectional associations between self-perceptions of aging and processing speed across 3 years. GeroPsych 2017; 30: 49-59.

354. Settin JM. Clinical judgement in geropsychology practice. Psychotherapy 1982; 19: 397-404.

355. Shah S, Cook DG. Inequalities in the treatment and control of hypertension: age, social isolation and lifestyle are more important than economic circumstances. J Hypertens 2001; 19: 1333-40.

356. Shippee T, Schafer M, Shippee N, Rinaldo L. Long-term effects of age discrimination on mental health: The role of perceived financial hardship. J Gerontol B Psychol Sci Soc Sci 2017; published online March 14. DOI: 10.1093/geronb/gbx017.

357. Silliman RA, Guadagnoli E, Weitberg AB, Mor V. Age as a predictor of diagnostic and initial treatment intensity in newly diagnosed breast cancer patients. J Gerontol 1989; 44: M46-50.

358. Siminoff LA, Zhang A, Colabianchi N, Sturm CM, Shen Q. Factors that predict the referral of breast cancer patients onto clinical trials by their surgeons and medical oncologists. J Clin Oncol 2000; 18: 1203-11.

359. Singer MS. Applicant age and selection interview decisions: effect of information exposure on age discrimination in personnel selection. J Pers Psychol 1989; 42: 135-53.

360. Snape E, Redman T. Too old or too young? The impact of perceived age discrimination. Hum Resour Manage 2003; 13: 78-89.

361. Steinfeld AD, Diamond JJ, Hanks GE, Coia LR, Kramer S. Patient age as a factor in radiotherapy. Data from the patterns of care study. J Am Geriatr Soc 1989; 37: 335-38.

362. Steverink N, Westerhof GJ, Bode C, Dittmann-Kohli F. The personal experience of aging, individual resources, and subjective well-being. J Gerontol B Psychol Sci Soc Sci 2001; 56: P364-73.

363. Stewart TL, Chipperfield JG, Perry RP, Hamm JM. Attributing heart attack and stroke to "Old Age": implications for subsequent health outcomes among older adults. J Health Psychol 2016; 21: 40-49.

364. Stewart TL, Chipperfield JG, Perry RP, Weiner B. Attributing illness to 'old age:' consequences of a self-directed stereotype for health and mortality. Psychol Health 2012; 27: 881-97.

365. Stillman AE, Braitman LE, Grant RJ. Are critically ill older patients treated differently than similarly ill younger patients? West J Med 1998; 169: 162-65.

366. Stone PH, Thompson B, Anderson HV, et al. Influence of race, sex, and age on management of unstable angina and non-Q-wave myocardial infarction: the TIMI III registry. JAMA 1996; 275: 1104-12.

367. Stratton MA, Jordan AH, Harrison DL, Jacobs EW, Skaggs VJ. Disparities in the prevalence of medication therapy for hyperlipidemia in the elderly. Consult Pharm 2007; 22: 847-54.

368. Sun JK, Kim ES, Smith J. Positive self-perceptions of aging and lower rate of overnight hospitalization in the US population over Age 50. Psychosom Med 2017 ; 79: 81-90.

369. Sun JK, Smith J. Self-perceptions of aging and perceived barriers to care: reasons for health care delay. Gerontologist 2017 ; 57S2: S216-26.

370. Sutin AR, Stephan Y, Carretta H, Terracciano A. Perceived discrimination and physical, cognitive, and emotional health in older adulthood. Am J Geriatr Psychiatry 2015; 23: 171-79.

371. Sutter M, Perrin PB, Tabaac AR, Parsa L, Mickens M. Do ableism and ageism predict college students’ willingness to provide care for a family member with a chronic health condition? Stigma Health 2017; 2: 110-20.

372. Talarico L, Chen G, Pazdur R. Enrollment of elderly patients in clinical trials for cancer drug registration: a 7-year experience by the US Food and Drug Administration. J Clin Oncol 2004; 22: 4626-31.

373. Thake M, Lowry A. A systematic review of trends in the selective exclusion of older participant from randomised clinical trials. Arch Gerontol Geriatr 2017; 72: 99-102.

374. Top M, Eris H, Kabalcioglu F. Quality of life and attitudes toward aging among older women in Turkey. Affilia 2012; 27: 406-19.

375. Tovel H, Carmel S, Raveis VH. Relationships among self-perception of aging, physical functioning, and self-efficacy in late life. J Gerontol B Psychol Sci Soc Sci 2017; published online May 25. DOI: 10.1093/geronb/gbx056.

376. Tran CT, Laupacis A, Mamdani MM, Tu JV. Effect of age on the use of evidence-based therapies for acute myocardial infarction. Am Heart J 2004; 148: 834-41.

377. Trigg R, Watts S, Jones R, Tod A, Elliman R. Self-reported quality of life ratings of people with dementia: the role of attitudes to aging. Int Psychogeriatr 2012; 24: 1085-93.

378. Trimble EL, Carter CL, Cain D, Freidlin B, Ungerleider RS, Friedman MA. Representation of older patients in cancer treatment trials. Cancer 1994; 74: 2208-14.

379. Uncapher H, Arean PA. Physicians are less willing to treat suicidal ideation in older patients. J Am Geriatr Soc 2000; 48: 188-92.

380. Van Spall HG, Toren A, Kiss A, Fowler RA. Eligibility criteria of randomized controlled trials published in high-impact general medical journals: a systematic sampling review. JAMA 2007; 297: 1233-40.

381. Vauclair C-M, Marques S, Lima ML, Abrams D, Swift H, Bratt C. Perceived age discrimination as a mediator of the association between income inequality and older people's self-rated health in the European Region. J Gerontol B Psychol Sci Soc Sci 2015; 70: 901-12.

382. Viitasalo N, Nätti J. Perceived age discrimination at work and subsequent long-term sickness absence among Finnish Employees. J Occup Environ Med 2015; 57: 801-05.

383. Villiers-Tuthill A, Copley A, McGee H, Morgan K. The relationship of tobacco and alcohol use with ageing self-perceptions in older people in Ireland. BMC Public Health 2016; 16: 627.

384. Vitman A, Iecovich E, Alfasi N. Ageism and social integration of older adults in their neighborhoods in Israel. Gerontologist 2014; 54: 177-89.

385. Volpone SD, Avery DR. It’s self defense: how perceived discrimination promotes employee withdrawal. J Occup Health Psychol 2013; 18: 430-48.

386. von Hippel C, Kalokerinos EK, Henry JD. Stereotype threat among older employees: relationship with job attitudes and turnover intentions. Psychol Aging 2013; 28: 17-27.

387. Votron L, D'Hoore W, Swine C, Daisne JF, Scalliet P. The opinion of general practitioners on the treatment of prostate and breast cancer in elderly people: Results of a survey based on clinical models. Clin Oncol (R Coll Radiol) 2004; 16: 474-78.

388. Wang J, Kollias J, Boult M, et al. Patterns of surgical treatment for women with breast cancer in relation to age. Breast J 2010; 16: 60-65.

389. Wanner R, McDonald L. Ageism in the labor market: estimating earnings discrimination against older workers. J Gerontol 1983; 38: 738-44.

390. Warmoth K, Tarrant M, Abraham C, Lang IA. Relationship between perceptions of ageing and frailty in English older adults. Psychol Health Med 2017; 1-10.

391. Weaver WD, Litwin PE, Martin JS, et al. Effect of age on use of thrombolytic therapy and mortality in acute myocardial infarction. The MITI Project Group. J Am Coll Cardiol 1991; 18: 657-62.

392. Weiss EM, Maurer TJ. Age discrimination in personnel decisions: a reexamination. J Appl Soc Psychol 2004; 34: 1551-62.

393. Westerhof GJ, Whitbourne SK, Freeman GP. The aging self in a cultural context: the relation of conceptions of aging to identity processes and self-esteem in the United States and the Netherlands. J Gerontol B Psychol Sci Soc Sci 2012; 67: 52-60.

394. Whincup PH, Emberson JR, Lennon L, Walker M, Papacosta O, Thomson A. Low prevalence of lipid lowering drug use in older men with established coronary heart disease. Heart 2002; 88: 25-29.

395. Whiting RM. The effect of ageism on law enforcement response to elder mistreatment. PhD thesis, University of Utah, 2015.

396. Wiel E, Di Pompeo C, Segal N, et al. Age discrimination in out-of-hospital cardiac arrest care: a case-control study. Eur J Cardiovascular Nurs 2017; 17: 505-12.

397. Wight RG, LeBlanc AJ, Meyer IH, Harig FA. Internalized gay ageism, mattering, and depressive symptoms among midlife and older gay-identified men. Soc Sci Med 2015; 147: 200-08.

398. Williams D, Bennett K, Feely J. Evidence for an age and gender bias in the secondary prevention of ischaemic heart disease in primary care. Br J Clin Pharmacol 2003; 55: 604-08.

399. Williams DG. Ageism and experience bias in employment interviews. Master's thesis, University of Missouri-Kansas City, 2015.

400. Williams KN, Herman R, Gajewski B, Wilson K. Elderspeak communication: impact on dementia care. Am J Alzheimers Dis Other Demen 2009; 24: 11-20.

401. Wiseman D. Patient characteristics that impact healthcare resource allocation choices: relative impact of mental illness, age, and parental status. J Appl Soc Psychol 2007; 37: 2072-85.

402. Wolff JK, Schüz B, Ziegelmann JP, Warner LM, Wurm S. Short-term buffers, but long-term suffers? Differential effects of negative self-perceptions of aging following serious health events. J Gerontol B Psychol Sci Soc Sci 2017; 72: 408-14.

403. Wolff JK, Warner LM, Ziegelmann JP, Wurm S. What do targeting positive views on ageing add to a physical activity intervention in older adults? Results from a randomised controlled trial. Psychol Health 2014; 29: 915-32.

404. Woodard S, Nadella PC, Kotur L, Wilson J, Burak WE, Shapiro CL. Older women with breast carcinoma are less likely to receive adjuvant chemotherapy: evidence of possible age bias? Cancer 2003; 98: 1141-49.

405. Wurm S, Benyamini Y. Optimism buffers the detrimental effect of negative self-perceptions of ageing on physical and mental health. Psychol Health 2014; 29: 832-48.

406. Wurm S, Tesch-Romer C, Tomasik MJ. Longitudinal findings on aging-related cognitions, control beliefs, and health in later life. J Gerontol B Psychol Sci Soc Sci 2007; 62: 156-64.

407. Wurm S, Tomasik MJ, Tesch-Romer C. Serious health events and their impact on changes in subjective health and life satisfaction: the role of age and a positive view on ageing. Eur J Ageing 2008; 5: 117-27.

408. Wurm S, Tomasik MJ, Tesch-Romer C. On the importance of a positive view on ageing for physical exercise among middle-aged and older adults: cross-sectional and longitudinal findings. Psychol Health 2010; 25: 25-42.

409. Wurm S, Warner LM, Ziegelmann JP, Wolff JK, Schüz B. How do negative self-perceptions of aging become a self-fulfilling prophecy? Psychol Aging 2013; 28: 1088-97.

410. Yamada Y, Merz L, Kisvetrova H. Quality of life and comorbidity among older home care clients: role of positive attitudes toward aging. Qual Life Res 2015; 24: 1661-67.

411. Yamada Y, Sugisawa H, Sugihara Y, Shibata H. Factors relating to organizational commitment of older male employees in Japan. J Cross Cult Gerontol 2005 ; 20: 181-90.

412. Yan E, Tang CS. Proclivity to elder abuse: a community study on Hong Kong Chinese. J Interpers Violence 2003; 18: 999-1017.

413. Yechezkel R, Ayalon L. Social workers' attitudes towards intimate partner abuse in younger vs. older women. J Fam Violence 2013; 28: 381-91.

414. Yee KW, Pater JL, Pho L, Zee B, Siu LL. Enrollment of older patients in cancer treatment trials in Canada: why is age a barrier? J Clin Oncol 2003; 21: 1618-23.

415. Yeom HE. Symptoms, aging-stereotyped beliefs, and health-promoting behaviors of older women with and without osteoarthritis. Geriatr Nurs 2013; 34: 307-13.

416. Yeom HE. Association among ageing-related stereotypic beliefs, self-efficacy and health-promoting behaviors in elderly Korean adults. J Clin Nurs 2014 ; 23: 1365-73.

417. Yon Y, Anderson L, Lymburner J, et al. Is Ageism in university students associated with elder abuse? J Intergener Relatsh 2010; 8: 386-402.

418. Yonemori K, Hirakawa A, Komiyama N, et al. Participation of elderly patients in registration trials for oncology drug applications in Japan. Ann Oncol 2010 ; 21: 2112-8.

419. Yuan ASV. Perceived age discrimination and mental health. Soc Forces 2007; 86: 291-311.

420. Zhang J, Nancy Xiaonan Y, Zhang J, Zhou M. Age stereotypes, flexible goal adjustment, and well-being among Chinese older adults. Psychol Health Med 2018; 23: 210-15.

421. Zhao Y, Dupre ME, Qiu L, Gu D. Changes in perceived uselessness and risks for mortality: evidence from a National sample of older adults in China. BMC Public Health 2017; 17: 561

422. Zulman DM, Sussman JB, Chen X, Cigolle CT, Blaum CS, Hayward RA. Examining the evidence: a systematic review of the inclusion and analysis of older adults in randomized controlled trials. J Gen Intern Med 2011; 26: 783-90.
