# Supplementary material for: Global reach of ageism on older persons’ health: A systematic review
Source: PLoS One. 2020 Jan 15;15(1):e0220857. doi: 10.1371/journal.pone.0220857 (PMC6961830; doi:10.1371/journal.pone.0220857)
Supplement: S4 Table — (PDF) [file pone.0220857.s004.pdf]

S4. Characteristics of 422 Studies of Ageism and Health [a]

STUDY CHARACTERISTICS	Number Of Studies	% of Total Studies
**Continents of studies**		
Africa	1	0.3
Asia	28	6.6
Australia/Oceania	13	3.1
Europe	129	30.6
North America	201	47.6
South America	2	0.5
Global[b]	49	11.6
**Development level of study countries**[c]		
More-developed countries	357	94.0
Less-developed countries	23	6.0
**Publication years**		
Before 1999	78	18.5
Since 2000	344	81.5
**Type of publications**		
Peer-reviewed articles	386	91.5
Grey literature	36	8.5
**Language**		
English	419	99.3
Others	3	0.7
**Type of studies**		
Observational studies	288	68.2
Experimental studies	134	31.8
**Ageism measures**		
Perceived age discrimination	261	61.9
Age stereotypes	97	23.0
Self-perceptions of aging	64	15.2
**Study quality**[d]		
Good quality	317	75.1
Medium to low quality	105	24.9

	Number Of Studies	% of Total Studies
**TARGETS OF AGEISM**[e]		
**Average age**[f]		
< 74		80.8
≥ 75		19.2
**Gender**		
More men	94	39.3
More women	145	60.7
**Education**		
Majority attended high school or less[g]	75	71.4
Majority attended college or more	30	28.6
**Race/Ethnicity**		
More majority-group members[g]	99	91.7
More minority-group members	9	8.3
**TARGETERS OF AGEISM**[e]		
**Average age**[f]		
18–30	14	32.6
≥ 30	29	67.4
**Gender**		
More men	21	42.0
More women	29	58.0
**Education**		
Majority attended high school or less[g]	12	26.7
Majority attended college or more	33	73.3
**Race/Ethnicity**		
More majority-group members[g]	9	90.0
More minority-group members	1	10.0

[a] Numbers may not sum to total due to missing data, and percentages may not sum to 100% due to rounding. [b] Conducted in more than one continent. [c] Levels of development are categorized in accordance with the Dept. of Economics and Social Affairs, United Nations. [d] Good-quality studies are studies that were appraised with overall score of 7 and above based on our quality appraisal checklist. [e] The tabulations of targets and targeters' information are based on available data from studies that reported detailed study sample characteristics. [f] Average age uses either mean or median age of study samples, whichever reported. Targets' mean age may be less than 50 years old due to baseline age in longitudinal studies, or studies of older persons that had larger proportions of younger persons. [g] Majority is defined as more than 50% of participants in the study sample.
