# Supplementary material for: Global reach of ageism on older persons’ health: A systematic review
Source: PLoS One. 2020 Jan 15;15(1):e0220857. doi: 10.1371/journal.pone.0220857 (PMC6961830; doi:10.1371/journal.pone.0220857)
Supplement: S5 Table — (PDF) [file pone.0220857.s005.pdf]

S5. Sensitivity Analysis of Ageism Adversely Impacted Health Across Geography, Time, Study Characteristics, and Characteristics of Targets and Targeters in Good-Quality Studies [a, b]

	Significant Ageism Associations, % (n) [b]	Chi-square Values
**STUDY CHARACTERISTICS**		
**Continents of studies**		
Africa	100.0 (1)	47.44***
Asia	94.6 (53)	
Australia/Oceania	96.9 (31)	
Europe	77.5 (182)	
North America	63.5 (339)	
South America	100.0 (1)	
Global[c]	76.9 (50)	
**Development level of study countries[d]**		
More-developed countries	70.1 (620)	7.38
Less-developed countries	90.0 (36)	
**Publication years**		
Before 1999	53.3 (163)	69.08***
Since 2000	79.6 (493)	
**Type of publications**		
Peer-reviewed articles	71.3 (617)	0.71
Grey literature	66.1 (39)	
**Language**		
English	70.7 (650)	0.83
Others	100.0 (2)	
**Type of studies**		
Observational studies	71.3 (506)	0.18
Experimental studies	69.8 (150)	
**Ageism measures**		
Perceived ageism	64.0 (403)	48.74***
Age stereotypes	82.5 (151)	
Self-perceptions of aging	91.1 (102)	

	Significant Ageism Associations, % (n) [b]	Chi-square Values
**TARGETS OF AGEISM[e]**		
**Average age[f]**		
< 74	69.7 (232)	4.81*
≥ 75	83.9 (47)	
**Gender**		
More men	76.2 (263)	0.03
More women	76.9 (173)	
**Education**		
Majority attended high school or less[g]	89.3 (117)	15.10***
Majority attended college or more	67.5 (52)	
**Race/Ethnicity**		
More majority-group members[g]	70.9 (180)	0.01
More minority-group members	72.0 (18)	
**TARGETERS OF AGEISM[e]**		
**Average age[f]**		
18–30	86.7 (13)	1.78
≥ 30	69.7 (46)	
**Gender**		
More men	68.4 (39)*	4.99*
More women	90.0 (27)	
**Education**		
Majority attended high school or less[g]	84.6 (11)	3.66
Majority attended college or more	55.8 (29)	
**Race/Ethnicity**		
More majority-group members[g]	67.7 (23)	0.47
More minority-group members	100.0 (1)	

*p<.05, **p<.01, ***p<.001.

[a] Good-quality studies are studies that were appraised with overall score of 7 and above based on our quality appraisal checklists. The good-quality studies (n=317) included 925 associations in total. [b] Numbers may not sum to total due to missing data, and percentages may not sum to 100% due to rounding. [c] Conducted in more than one continent. [d] Levels of development are categorized in accordance with the Dept of Economic and Social Affairs, United Nations. [e] The tabulations of targets and targeters' information are based on available data from studies that reported detailed study sample characteristics. [f] Average age uses either mean or median age of study samples, whichever reported. Targets' average age may be less than 50 years old due to baseline age in longitudinal studies, or studies of older persons that had larger proportions of younger persons. [g] Majority is defined as more than 50% of participants in the study sample.
