# Supplementary material for: Global reach of ageism on older persons’ health: A systematic review
Source: PLoS One. 2020 Jan 15;15(1):e0220857. doi: 10.1371/journal.pone.0220857 (PMC6961830; doi:10.1371/journal.pone.0220857)
Supplement: S6 Table — (PDF) [file pone.0220857.s006.pdf]

S6. Sensitivity Analysis of Predictors of Significant Ageism-Health Associations Remained the Same after Adjusting for Study Sample Size [a,b]

	Multivariate Analysis Adjusted for Study Sample Size	
	**Total Associations (n=1,159)**	**Total Associations in Good-quality Studies (n=925)[c]**
	OR (95% CI)	OR (95% CI)
**STUDY CHARACTERISTICS**		
**Continents of studies**		
North America [d]	1.0	1.0
Other Continents	2.5 (1.89-3.23)***	2.44 (1.82-3.33)***
**Development level of study countries[a]**		
More-developed countries	1.0	1.0
Less-developed countries	5.15 (2.06–12.99)***	6.85 (2.11–12.22)**
**Publication years**		
Before 1999	1.0	1.0
Since 2000	3.28 (2.48–4.33)***	3.38 (2.50–4.56)***
**Type of publications**		
Peer-reviewed articles	1.0	1.0
Grey literature	0.75 (0.46–1.25)	0.75 (0.43–1.33)
**Language**		
English	1.0	1.0
Others	3.45 (0.43-25.0)	<0.00 (<0.00- >999.99)[b]
**Type of studies**		
Observational studies	1.0	1.0
Experimental studies	1.08 (0.79-1.45)	0.85 (0.61-1.19)
**TARGETS OF AGEISM[e]**		
**Average age[f]**		
< 74	1.0	1.0
≥ 75	1.01 (0.99–1.04)	1.00 (1.00–1.01)
**Gender**		
More men	1.0	1.0
More women	1.03 (0.70–1.50)	.097 (0.65–1.47)
**Education**		
Majority attended high school or less[g]	1.0	1.0
Majority attended college or more	0.26 (0.13-0.53)***	0.24 (0.11-0.53)***
**Race/Ethnicity**		
More majority-group members	1.0	1.0
More minority-group members	1.05 (0.43–2.59)	0.96 (0.38–2.41)

**TARGETERS OF AGEISM**[e]		
**Average age**[f]		
≥ 30	1.0	1.0
18–30	1.00 (1.00–1.01)	1.00 (1.00–1.01)
**Gender**		
More women	1.0	1.0
More men	1.00 (1.00–1.01)	1.00 (1.00–1.01)
**Education**		
Majority attended high school or less[g]	1.0	1.0
Majority attended college or more	1.00 (1.00–1.01)	1.00 (1.00–1.01)
**Race/Ethnicity**		
More minority-group members	1.0	1.0
More majority-group members	<0.00 (<0.00–>999.99)[b]	<0.00 (<0.00–>999.99)[b]

*p<.05, **p<.01, ***p<.001.

[a] Levels of development are categorized in accordance with the Dept of Economic and Social Affairs, United Nations. [b] Cell number too small to provide exact OR estimates. [c] Good-quality studies are studies that were appraised with overall score of 7 and above based on our quality appraisal checklists. The good-quality studies (n=317) included 925 associations in total. [d] North America was chosen as reference group because it is the continent that has the largest number of studies. [e] The tabulations of targets and targeters' information are based on available data from studies that reported detailed study sample characteristics. [f] Average age uses either mean or median age of study samples, whichever reported. Targets' average age may be less than 50 years old due to baseline age in longitudinal studies, or studies of older persons that had larger proportions of younger persons. [g] Majority is defined as more than 50% of participants in the study sample.
