# Supplementary material for: Global reach of ageism on older persons’ health: A systematic review
Source: PLoS One. 2020 Jan 15;15(1):e0220857. doi: 10.1371/journal.pone.0220857 (PMC6961830; doi:10.1371/journal.pone.0220857)
Supplement: S1 Appendix — (PDF) [file pone.0220857.s007.pdf]

S1 Appendix.

Calculations of Global Depression Cases due to Ageism in Older Persons

We calculated the number of depression cases due to ageism (i.e., age discrimination, negative age stereotypes, and negative self-perceptions of aging) for all persons aged 50 years or older in more-developed and less-developed countries during one year. As we were not able to model the impact of ageism across our 11 health domains because of heterogeneity of outcomes and measures, we modeled the impact of ageism on one outcome to exemplify the potential harm of ageism. We selected depression because it was the largest category of homogeneous studies included in our review with respect to outcome measure; 14 out of 17 studies utilized the Center for Epidemiologic Studies Depression Scale (CESD) [1]. As part of the method that we will explain toward the end of this description, because the predictors are interrelated, we took the conservative approach of removing the overlap of the influence of the three ageism predictors on the outcome of later life depression.

In order to calculate this estimate, we drew upon a method reported earlier [2] and literature to obtain four sets of estimates for calculation, including prevalence of ageism predictors among older persons, prevalence of depression among older persons, total global population of older persons, and the effects of ageism on depression among older persons based on our meta-analysis of 17 studies included in the present systematic review.

The first estimate, the prevalence of three ageism predictors, were drawn from conservative ageism calculations in an earlier paper [2]. Using this approach, the prevalence of age discrimination and negative self-perceptions of aging based on the Health and Retirement Study was 28.02% for age discrimination, and 21.09% for negative self-perceptions of aging. The prevalence of negative age stereotypes, based on the Precipitating Events Project, was

60.25%. These three estimates were used as prevalence of ageism for both more-developed and less-developed regions, assuming the prevalence of ageism were consistent across more- and less-developed regions. This is a conservative assumption because the prevalence of ageism has been reported to be higher in less-developed countries compared to more-developed countries [3].

The second estimate, prevalence of one-year depression in older persons, was drawn from a study that reported the one-year prevalence of depression in persons aged 50 - 64 and 65 and older in more-developed and less-developed regions, based on the WHO World Mental Health Surveys [4]. The WHO World Mental Health Consortium includes nationally or regionally representative surveys that represent all regions of the world, and with estimated sample sizes over 155,000 individuals. Depression was assessed by the WHO Composite International Diagnostic Interview (CIDI) Version 3.0, a fully structured diagnostic interview [5]. Overall, this study found that in the more-developed countries, 5.1% of those 50 - 64 and 2.6% of persons 65 and older experienced depression, whereas 6.8% of those 50 - 64 and 7.5% persons 65 and older experienced depression in the less-developed countries.

The third estimate, population estimates of persons aged 50 and over, was drawn from the estimates from Population Bureau of United Nation [6]. We used the population estimates of persons aged 50 - 64, and 65 and over, in more-developed countries and less-developed countries in the year 2015. Overall, 251,960,000 persons aged 50 - 64 and 220,572,000 persons aged 65 and over resided in more-developed countries; 785,616,000 persons aged 50 - 64 and 391,325,000 persons aged 65 and over resided in less-developed countries.

The last estimate used in the calculation was the effect sizes of ageism on depression based on a random-effect meta-analysis. We combined the 17 studies included in the review that

assessed depression as outcome. Overall, the effect size of age discrimination on depression was 0.07 [95% CI= (0.04, 0.09)], for the negative age stereotypes on depression it was 0.13 [95% CI= (0.05, 0.21)]; and for the self-perceptions of aging on depression it was 0.07 [95% CI= (0.06, 0.08)].

To demonstrate how we calculated the number of depression cases due to three ageism predictors in persons aged 50 and over in more- and less-developed countries, the following example illustrates the contribution of negative self-perceptions of aging due to depression for persons aged 50 - 64 in the less-developed countries (The same approach was used across the estimations for the combination of three ageism predictor, i.e., age discrimination, negative age stereotypes, and negative self-perceptions of aging; two age groups, i.e., 50 - 64 years old, and 65 and more; and two regions, i.e., more-developed countries, and less-developed countries). We took the following steps to calculate this number. Step one, to calculate the total number of older persons aged 50 - 64 in the positive-self-perception-of-aging and negative-self-perception-of-ageing groups, we multiplied the number of older persons aged 50-64 in less-developed countries. Step two, to calculate the number of individuals in the positive-self-perception-of-aging and negative-self-perception-of-ageing groups who experienced depression, we used the number of older persons aged 50 - 64 who experienced depression in the less-developed countries [4] and the negative-self-perception-of-aging relative risk of experiencing depression compared with positive-self-perception-of-aging relative risk (derived from the effect sizes of ageism on depression from the systematic review), and then the number of persons in the self-perception-of-aging groups (from step one). Specifically, we converted the effect size, the standardized mean difference (SMD) to odds ratio (OR) using the formula $SMD = \frac{\sqrt{3}}{\pi}\, lnO$ [7]. We then derived the relative risk from OR using the approach proposed by Zhang et al [8]. Step

three, to calculate the excess prevalence of depression due to negative-self-perceptions-of-aging, we subtracted the positive-self-perceptions-of-aging- depression prevalence from the negative-self-perceptions-of-aging- depression prevalence. Step four, we calculated the excess cases of depression due to negative-self-perceptions-of-aging, to multiple excess prevalence (step three) by number of people in the negative-self-perceptions-of-aging group (step one).

To generate the non-overlapping depression cases due to ageism, we added the depression cases of three ageism predictors and removed the overlapping cases of depression of the three ageism predictors because the three predictors are correlated. Following an earlier methodology to remove overlap and identification of shared variance of the ageism predictors [2], we entered into our model the shared common variance of the age stereotype and self-perceptions-of-aging measures as R^2=.23; for the self-perception of aging measure and age discrimination measure as R^2=.16; and for the age stereotype and age discrimination measures as R^2=.19.

To remove duplicate cases due to the overlap of three ageism predictors using the shared common variance, we began with negative age stereotypes that generated the greatest number of depression cases, 3,783,958. We then calculated the additional non-over-lapping cases due to age discrimination, which is (1-.19) or .81 of the total number of cases associated with age discrimination, i.e., .81 of 1,952,802 or 1,581,770, and the additional non-over-lapping cases due to negative self-perceptions of aging which is 1,494,523* (1-.23)(1-.16)=966,657. Thus, according to this final model, there were 6,332,385 excess cases of depression due to ageism, with 831,041 in more-developed countries and 5,565,428 in less developing countries.

These estimates are conservative calculations due to the following reasons. First, as aforementioned, the prevalence of ageism tends to be higher in less-developed countries than

more-developed countries and our review found that the prevalence of significant ageism-health studies tends to be higher in less developed than more developed countries [3]. Thus, by applying the same estimates of ageism prevalence from more-developed to less developed countries, this is likely an underestimation of the ageism prevalence and its impact in the less-developed countries. Second, as many cases of depression in older persons are misconstrued as a normal part of aging [9], the prevalence of depression may be under-estimated in the WHO Mental Health Survey Initiative [4]. Third, we removed the overlap of the influence of the three ageism predictors on depression.

Several assumptions that went into the calculation of the number of depression cases due to ageism should be kept in mind. First, when we conducted the meta-analysis to produce the combined effect size of the ageism-depression studies we found that even though 14 of 17 studies use the same outcome measure, high heterogeneity was still found with an I-squared statistic of 89% [7]. This is likely due in part to the different predictor measures, years of study, locations and target characteristics. Second, we made an assumption about the number of older people living in the different parts of the world based on 2015 numbers from the UN so the number of cases would be lower if extrapolated to an earlier year but higher if extrapolated to a more recent year. Third, the ageism-depression studies were based on a number of observational studies rather than randomized clinical trials. However, in this model we were able to examine the impact of ageism above and beyond effects of age and sex because the individual studies of the impact of ageism on depression adjusted for these factors as covariates in the multivariate models that produced the effect sizes that we used in our overall estimate of the impact of ageism on depression. In addition, we conducted a stratified model that took into account age differences in depression.

Supporting our systematic review of the injurious reach of ageism, this secondary analysis provides additional quantitative evidence of ageism's impact on the health of older persons.

Reference List

1. Radloff LS. The CES-D scale: A self-report depression scale for research in the general population. Appl Psychol Meas. 1977; 1:385-401.

2. Levy BR, Slade MD, Chang ES, Kannoth S, Wang SY. Ageism amplifies cost and prevalence of health conditions. Gerontologist 2018; published online November 13. DOI: 10.1093/geront/gny131.

3. Nash P, Officer A, de la Fuente-Nunez V, Schneiders M, Thiyagarajan J. Global trends in ageist disposition: comparing low, middle, and high income countries using the World Values Survey Wave 6. Innov Aging. 2018; 2:34.

4. Kessler RC, Birnbaum HG, Shahly V, Bromet E, Hwang I, McLaughlin KA, et al. Age differences in the prevalence and co-morbidity of DSM-IV major depressive episodes: results from the WHO World Mental Health Survey Initiative. Depress Anxiety. 2010;27(4):351-64.

5. Kessler RC, Ustun TB. The World Mental Health (WMH) Survey Initiative Version of the World Health Organization (WHO) Composite International Diagnostic Interview (CIDI). Int J Methods Psychiatr Res. 2004;13(2):93-121.

6. United Nationas Population Division. World Population Prosepects 2017 Data Query. New York: United Nationas Population Division, 2017.

7. Higgins J, Green S. Cochrane handbook for systematic reviews for interventions, version 5.1.0. London: The Cochrane Collaboration, 2011.

8. Zhang J, Yu KF. What's the relative risk? A method of correcting the odds ratio in cohort studies of common outcomes. JAMA. 1998;280(19):1690-1.

9. Haigh EAP, Bogucki OE, Sigmon ST, Blazer DG. Depression among older adults: A 20-year update on five common myths and misconceptions. Am J Geriatr Psychiatry. 2018; 26(1):107-22.
